# A Systematic Review of Research Gaps in the Built Environment of Inpatient Healthcare Settings

**DOI:** 10.1177/19375867241251830

**Published:** 2024-05-28

**Authors:** Marie Elf, Ruby Lipson-Smith, Maya Kylén, Juan Pablo Saa, Jodi Sturge, Elke Miedema, Susanna Nordin, Julie Bernhardt, Anna Anåker

**Affiliations:** 1 School of Health and Welfare, Dalarna University, Falun, Sweden; 2 The Florey Institute of Neuroscience and Mental Health, University of Melbourne, VIC, Australia; 3 The MARCS Institute for Brain, Behaviour and Development, Western Sydney University, Westmead, NSW Australia; 4 Department of Health Sciences, Lund University, Lund, Sweden; 5 School of Allied Health, Human Services and Sport, La Trobe University, Bundoora, Melbourne, VIC, Australia; 6 Department of Design, Production and Management, Faculty of Engineering Technology, University of Twente, The Netherlands; 7 InHolland University of Applied Science, Domain Technology, Design and Computation, Division of Built Environment, The Netherlands

**Keywords:** built environment, evidence-based design, evidence-gap map, hospital, healthcare architecture, healthcare settings, systematic review

## Abstract

**Objective:**

This study utilized the evidence-gap map method and critically examined the scope, methodologies, and focus of the studies that investigated the influence of the built environment on inpatient healthcare settings over a decade (2010–2021).

**Methods:**

We conducted a systematic review per the preferred reporting items for systematic reviews and meta-analyses guidelines and surveyed 406 articles, primarily from North America and Europe.

**Results:**

Our findings revealed a dominant focus on architectural features (73%), such as room design and ward layout. Comparatively, there was less emphasis on interior-, ambient-, social-, and nature-related features. Most previous studies explored multiple environmental features, which indicated the intricacy of this field. Research outcomes were diverse, with person-centered care (PCC) being the most frequently investigated, followed by safe care, emotional well-being, activity, and behavior. Furthermore, research methods varied considerably based on the study’s outcomes and features. Clinical outcomes and safe care favored quantitative methods, activity and behavior favored mixed methods, and PCC favored qualitative research.

**Conclusion:**

This review provides an in-depth overview of the existing studies on healthcare design research and sheds light on the current trends and methodological choices. The insights garnered can guide future research, policy-making, and the development of healthcare facilities.

This is a systematic review of research gaps in the built environment of inpatient healthcare settings. The importance of design features in healthcare environments to improve the health, safety, and well-being of patients, staff, and relatives, as well as to improve the efficiency of healthcare services, is increasingly recognized (Bernhardt et al., 2022; Kevdzija & Marquardt, 2022; Rosbergen et al., 2022, World Health Organization, 2023) and the use of up-to-date evidence in planning and designing healthcare environments (Hall et al., 2017; Hamilton, 2023). The influential work of Roger Ulrich and colleagues (Ulrich, 1984; Ulrich et al., 2008) has been instrumental in shaping the understanding of how healthcare environments promote health and well-being. They identified critical design elements such as single rooms, pleasant atmosphere, natural sunlight, views of nature, ease of navigation, and personal environmental control, linking these to improved health outcomes (Ulrich, 2008; Ulrich et al., 2012).

Recent reviews of the literature have extended these findings and summarized the evidence regarding how various design features such as layout, sound, light, and nature affect patient, staff, and visitor health and well-being in both acute and subacute care contexts (Bernhardt et al., 2022; Brambilla et al., 2019; Drahota et al., 2012; Huisman et al., 2012; Lipson-Smith et al., 2021; Shannon et al., 2020a). These elements have been shown to reduce stress and anxiety and improve staff collaboration. Dijkstra et al. (2006) and Simonsen et al. (2022) demonstrated the positive effects of natural light and greenery on improved patient recovery and reduced hospital length of stay. Studies reported have similarly found that evidence-grounded environmental features impact behavior and well-being (Eijkelenboom & Bluyssen 2022; Verderber et al., 2021). Moreover, recent literature reviews have brought attention to specific environmental factors, such as noise levels (de Lima Andrade et al., 2021) and heating, ventilation, and air conditioning systems (Salonen, 2013; Shajahan et al., 2019). Some design features that are promoted in some clinical contexts, however, have less of an evidence base in other settings. Single-patient rooms, for example, are supported by evidence in acute care (Taylor et al., 2018), but in specific clinical settings, such as rehabilitation environments, have been associated with falls risk, inactivity, social isolation, and reduced interaction with staff (Anåker et al., 2022; Anåker et al., 2019). A recent review recommends a multidimensional approach to studying falls in healthcare facilities, considering the organization, people, and environment (Taylor & Hignett, 2016). Reviews have also highlighted the need for more operationalization of concepts related to the built environment and consistent definitions and applications of variables (Anåker et al., 2017), for example, in health promotion studies (Miedema, 2022). Furthermore, a review highlighted the value of applying theoretical frameworks in built environment research to better understand the impact of built environments on different outcomes, such as health, social interaction, or person-centered care (PCC); (Shannon et al., 2020b). Another review suggested that better evaluation of the impact of built environments on physical health, mental well-being, and social relationships requires improvement and validation of outcome measurement tools used in built environment research (Elf et al., 2017; Kylén et al., 2022).

These reviews typically concentrate on a specific setting, outcome, or population. Thus, there is a noticeable need since Ulrich et al. (2008) review to simultaneously examine a wide range of settings, outcomes, populations, and design characteristics. Undertaking such a study could offer a more holistic understanding of how these components are connected. It would also provide insights into which elements, that is, environments, populations, outcomes, or methods, are being investigated separately or in combination. This broad perspective has not been achieved with the current, more narrowly focused reviews, although they undoubtedly have their relevance.

In the intricate and evolving field of healthcare environment design, a comprehensive review focused on gaps in knowledge is important for several key reasons. Healthcare environments are characterized by their intricate interplay between natural (e.g., green and blue), built (e.g., spaces and objects), and social factors (e.g., models of care, values, and quality of relationships); (McCormack & McCance, 2006; Miedema, 2020). An evidence-gap study systematically identifies areas not sufficiently explored in the existing research. This can include specific design elements, patient populations, or outcomes that have not been adequately addressed. Such a review would provide a clear direction for future research efforts by defining these gaps, thereby ensuring that subsequent research is both focused and relevant to the emerging needs within healthcare settings.

Hamilton (2014, 2023) and Halawa et al. (2020) advocate that evidence-based design relies on comprehensive, up-to-date information. An evidence-gap study contributes to this by highlighting where current evidence is lacking, allowing designers and architects to base their decisions on a more complete understanding of the field. The healthcare sector continuously evolves with new care models, technological advancements, and patient demographics. An evidence-gap study helps ensure design practices keep pace with these changes by identifying areas where additional research can inform more adaptive and responsive design strategies.

Such knowledge is critical to inform healthcare designers, policymakers, and to guide researchers in their future work. We also recognize the value for students to have an updated review. Our review focuses on inpatient care, which offers insights applicable to broader healthcare contexts. Inpatient settings involve significant resource organization, multidisciplinary teamwork, and provide a detailed view of patient outcomes and quality of care. Understanding inpatient care is crucial for adapting to the evolving healthcare landscape.

We used the evidence-gap map (EGM) method (Snilstveit et al., 2013; White et al., 2020), a systematic approach to summarizing and visualizing published evidence in a specific field while identifying research gaps. The method aims to facilitate evidence-based decision-making by presenting user-friendly information.

In this review, we provide a descriptive exploration of the current state of research concerning the built environment in inpatient healthcare settings. The study is organized around four central themes:

1. What healthcare settings have been the focus of the studies included?
2. What design features of the built environment have been predominantly examined?
3. What health-related outcomes have been explored in association with design features?
4. What research designs and methods have been employed to investigate these associations?

## Method

The systematic review was based on the EGM method (Snilstveit et al., 2013) and followed a five-stage process aligned with the Campbell Collaboration guidance (White et al., 2020): (1) define a framework; (2) identify the available research; (3) appraise its quality; (4) extract, code, and summarize the data; and (5) visualize and present the findings in a user-friendly manner.

An interdisciplinary team of researchers from nursing, architecture, medicine, occupational therapy, psychology, and geography conducted a comprehensive review from 2010 to 2022, which covers a time soon after publication of Ulrich et al. (2008) seminal review to present day. This period also captures a time of significant acceleration in built environment research. The study adhered to the preferred reporting items for systematic reviews and meta-analyses (PRISMA) guidelines (Moher et al., 2011). The protocol was published in Nursing Open (Elf et al., 2020) and registered with the International Prospective Registry of Systematic Studies (CRD42020126905). The protocol article included additional objectives that examined the impact of the physical environment on different health-related outcomes. These will be addressed and published in subsequent review articles.

### Framework and Scope

**Figure 1.**
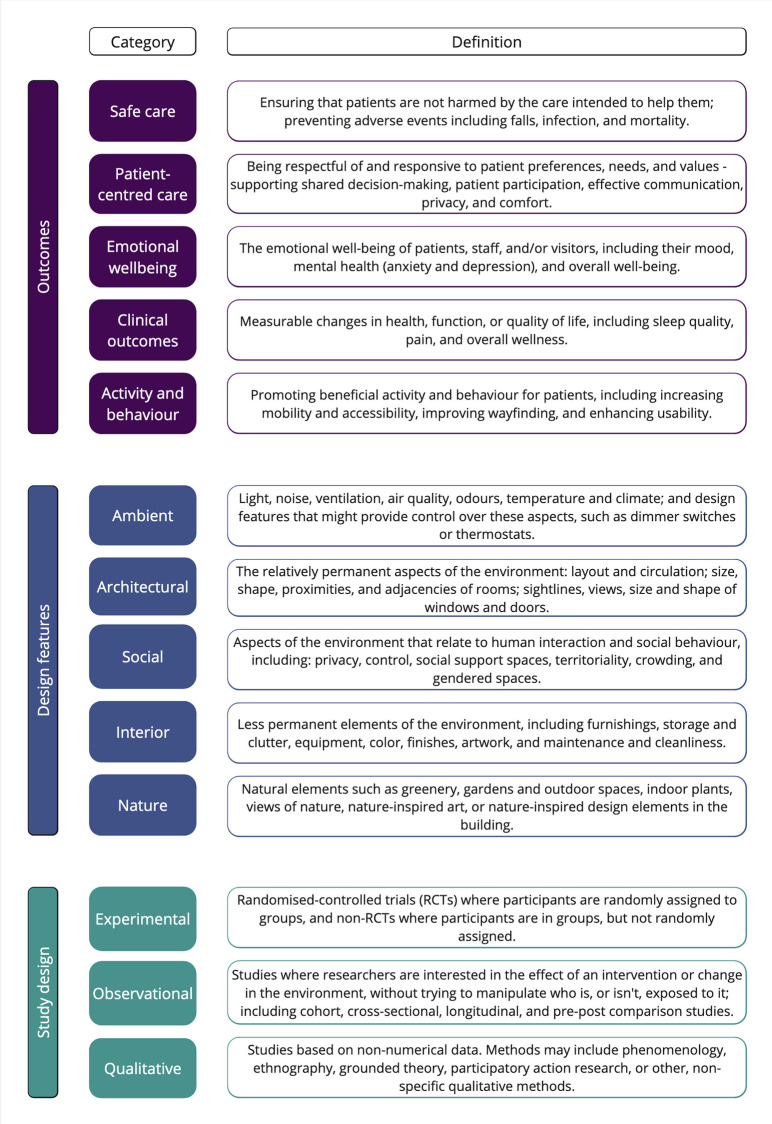
Review framework.

Figure 1 presents the framework of this review. The outcomes, design features, and study design have been outlined. To define the outcomes of interest at the prescreening stage, we referred to the Institute of Medicine framework (*Swedish National Board of Health and Welfare, 2016*; Wolfe, 2001), which emphasized the importance of the built environment in achieving high-quality standards and safe care, and a recent literature review of evidence-based healthcare design research (Lipson-Smith et al., 2021). The IOM framework includes six well-established indicators: safety, timeliness, effectiveness, efficiency, equity, and patient-centeredness. Guided by these indicators, we defined the following outcomes: safe care, person-centered care, emotional well-being, clinical outcomes, activities and behaviors. Our definition of environmental features was adapted from Harris’s model (Harris et al., 2002), which included four categories of built-environment features: ambient, architectural, social, and interior. In addition, a fifth category, “nature,” was added to address the impact of nature and the outdoors in healthcare design.

### Identifying Available Research

Eligibility criteria were based on the problem, intervention, comparison, and outcome framework (Eriksen & Frandsen, 2018), as outlined in Table 1. All articles examined the impact of the built environment in inpatient healthcare settings, were written in English, and published in peer-reviewed journals between 2010 and 2022. We excluded reviews and studies that focused on outpatient care and planning and design processes according to the exclusion criteria in Table 1.

**Table 1.** PICO applied.

Problem, Intervention, Comparison, and Outcome	Description	Exclusions
Population (P)	Persons (adults and children) with health conditions treated at healthcare environments, as well as significant others and staff	We excluded articles focused on outpatient and residential care facilities
Interventions (I)	All studies that analyzed the impact of the built environment of healthcare environments on their users (i.e., patients, significant others, staff; studies that can relate outcomes to specific aspects of the built environment)	We excluded literature reviews, discussion or theoretical papers, articles on the design process and future design plans, simulation studies, environmental measurements without human response, editorials, and instrument development studies. In addition, we excluded papers where the built environment was a secondary finding and not mentioned as the study’s primary aim
Comparison (C)	Not applicable or all kind of studies, such as cross-sectional, randomized controlled trial, and descriptive.	
Outcomes (O)	Quantitative and qualitative measures that reflect the built environment and its association to Institute of Medicines six domains of healthcare quality: safe, effective, patient-centered, timely, efficient, and equitable	

Research methods and designs were classified into three main categories: quantitative methods (measures and self-reported instruments), qualitative methods (interviews and observations), and mixed methods (combined quantitative and qualitative methods; Rutberg & Bouikidis, 2018; Swedish Agency for Health Technology Assessment and Assessment of Social Services [SBU], 2017).

### Database Search

The research team developed a comprehensive search strategy using MeSH and index terms in consultation with an information specialist from Chalmers University of Technology. We divided the search terms into three blocks and required that an article’s title or abstract contains at least one term from each block to be included. We used the search terms that were mesh terms in respective database. Block 1—Physical Environment: This block ensured that articles focused on the physical aspects of the environment. Including synonyms and close terms broadened the search to capture relevant articles that might have used different terminologies. Block 2—Participants: We included terms like “patient,” “family,” “visitors,” and “caregivers” (along with their synonyms and close words) to ensure that research involving human subjects in care environments was captured. Block 3—Care Environments: This block focused on the care setting (like various units, hospitals, nursing homes, home care, etc.). The Boolean table is added (Additional File 1).

**Figure 2.**
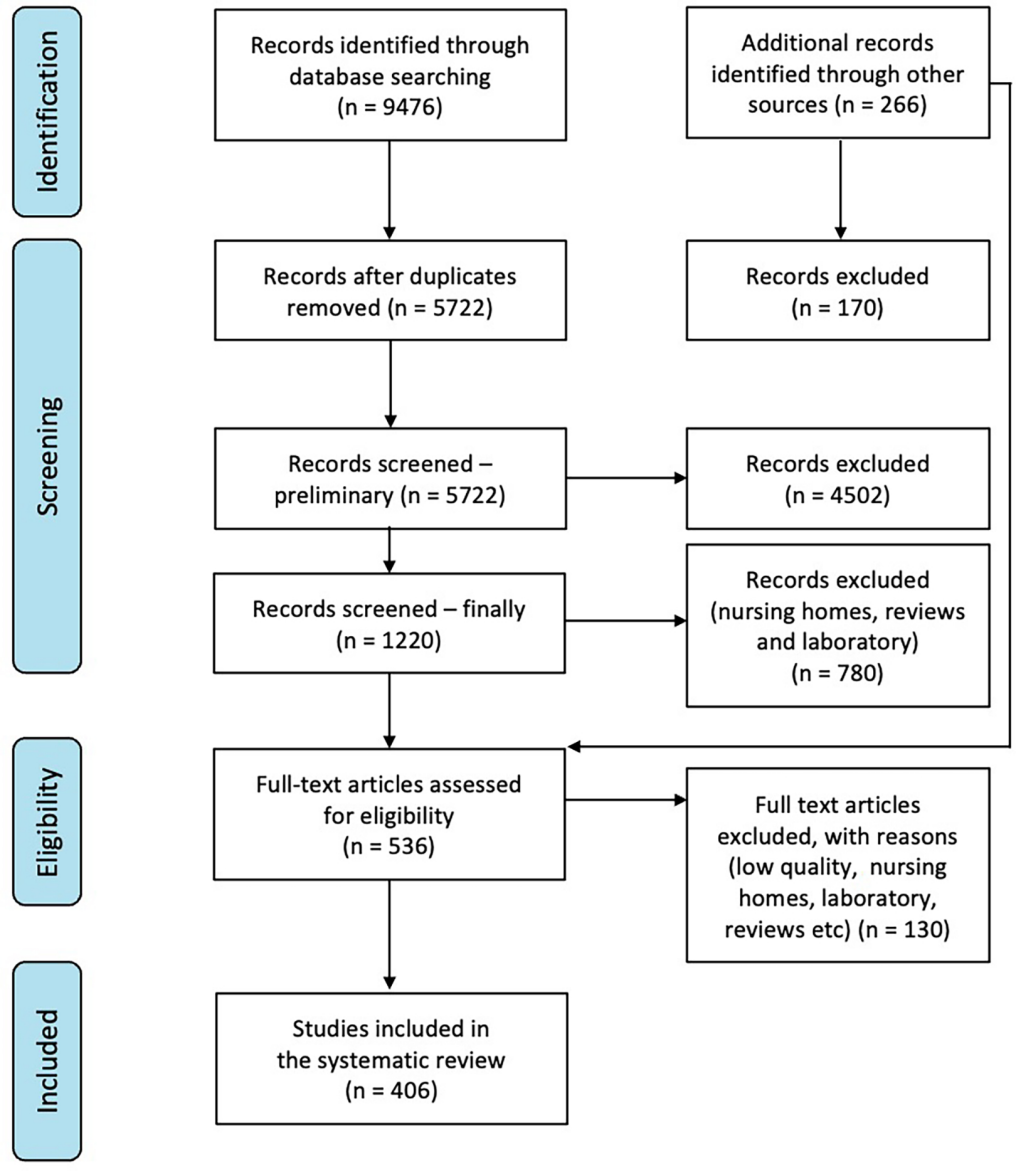
Preferred reporting items for systematic reviews and meta-analyses (PRISMA).

Five databases, PubMed, Cochrane Library, CINAHL, Web of Science, and Scopus, were searched for articles published from 2010 to 2021. Two researchers searched and assessed the strategy against a list of known and relevant articles. Reference lists of included articles were manually searched for additional relevant articles. Figure 2 shows the PRISMA flowchart.

### Screening and Selection

All the identified articles were merged, and duplicates were removed. They were uploaded to EndNote and a shared Excel document. Titles and abstracts were screened by five reviewers. A minimum of two reviewers per article met the predefined study eligibility criteria. Potentially eligible articles were separately screened by three reviewers, and conflicts were resolved through discussion, and reasons to exclude the articles were recorded.

### Quality Appraisal

The quality of the data sources was appraised according to the SBU (2017). Three of the authors split the articles between them and independently assessed the quality of eligible articles. A randomly selected sample was drawn for the calibration of the assessment among the authors. This step helped to align the evaluation criteria and approach. In instances of disagreement regarding the quality rating of an article, the authors engaged in discussion and conducted further evaluations until a consensus was reached. The eligible articles were then categorized into three quality levels a method also utilized in other studies: low (less than 49%), medium (50%–79%), and high (over 80%) quality. All papers included in the final review were assessed to have a quality above 50%.

### Data Extraction and Management

To extract and verify the data independently, we employed a predefined data collection form in Microsoft Excel (Additional File 2). The form contained crucial study characteristics, which included the authors, aim(s), study population, study design, design features, and outcomes. When extracting the data, authors summarized the research focus and outcomes of the included studies and then grouped these into categories, which were reviewed and refined by all authors until consensus was reached on a final five categories that encapsulated all the types of outcomes investigated in the included articles. In many cases, a single article addressed multiple populations, outcomes, and design features. For example, some studies included patients and staff or investigated social and architectural features together. Consequently, articles were categorized based on all their relevant study characteristics. Any discrepancies were thoroughly addressed through discussion to ensure consistency and accuracy.

After the information was summarized based on relevant characteristics, data were systematically categorized, grouped, and analyzed. Data were presented via tables and figures to ensure a comprehensive and transparent overview of the evidence, which included potential gaps. Additionally, we provided a narrative synthesis to contextualize and interpret the results coherently and efficiently. This approach allowed us to identify patterns and relationships between study characteristics and outcomes and draw meaningful conclusions.

## Results

Our search strategy identified 9476 publications and 266 additional publications from other sources. After duplicates were removed and the titles and abstracts were screened, 536 publications were selected for full-text review (Figure 2). From these, 129 low-quality publications were excluded. Finally, 406 eligible publications were selected. This review resulted in an open access database that can be updated.

### General Characteristics of the Included Papers

The articles were published between 2010 and 2021, with the majority published after 2015 (*n* = 226, 56%). All had a quality rating of at least 50%, and 43 (11%) scored above 85%. Although 406 studies were included, many could be classified under multiple outcomes and design features.

**Figure 3.**
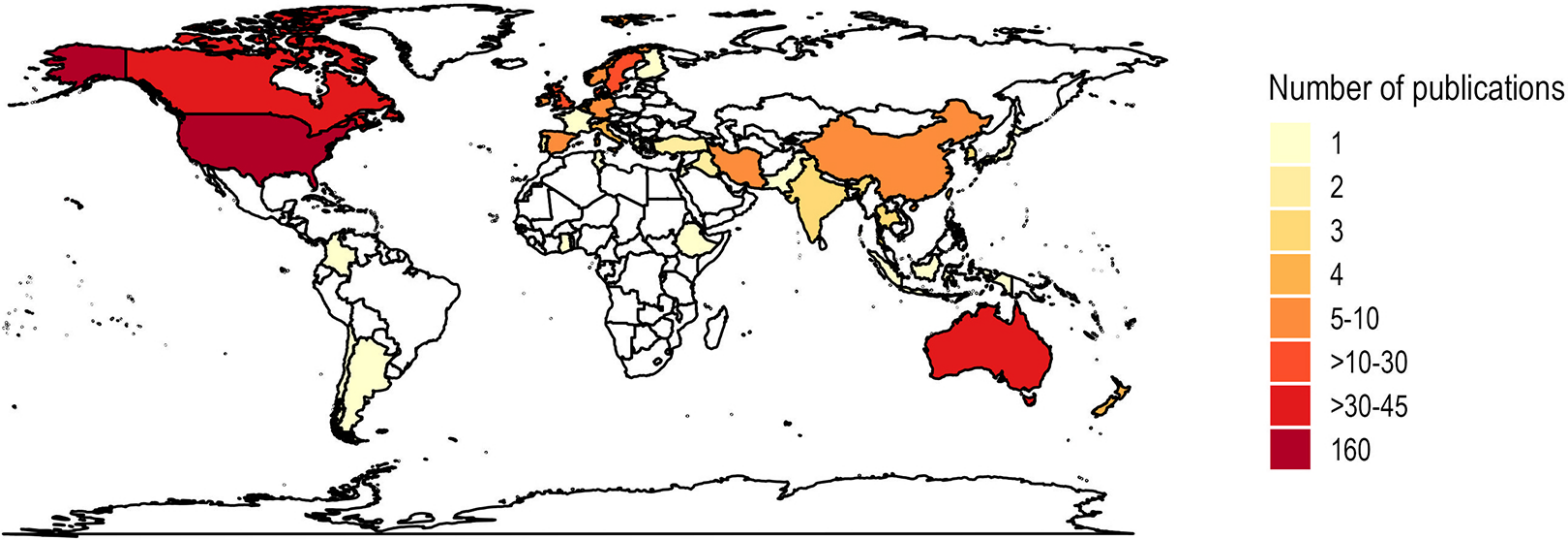
Geographical map.

Figure 3 shows the geographical distribution of the articles by country (i.e., where the study was conducted). Almost half were conducted in North America (*n* = 189, 46%), with the majority of these from the United States (*n* = 158, 84%). A total of 116 articles (29%) were from European countries, with over one-third of these (*n* = 42, 36%) being Scandinavian publications. Studies from Africa were the least common (*n* = 3, 1%) and were published since 2016.

The number of participants varied widely and ranged from just one participant in a study to over 1 million in another (retrospective study). Of the 406 studies, 30 (7%) did not report the number of participants.

Most studies reported on patients (*n* = 267, 66%) and those 187 (47%) addressed only patients; 195 (48%) papers addressed staff, from which 119 (29%) addressed only staff. Few addressed visitors (*n* = 54, 13%) from which 8 (2%) addressed only visitors. Some studies combined different types of participants (*n* = 90, 22%). In all papers, only 188 (48% of total) included participant numbers.

### Types of Healthcare Settings

**Figure 4.**
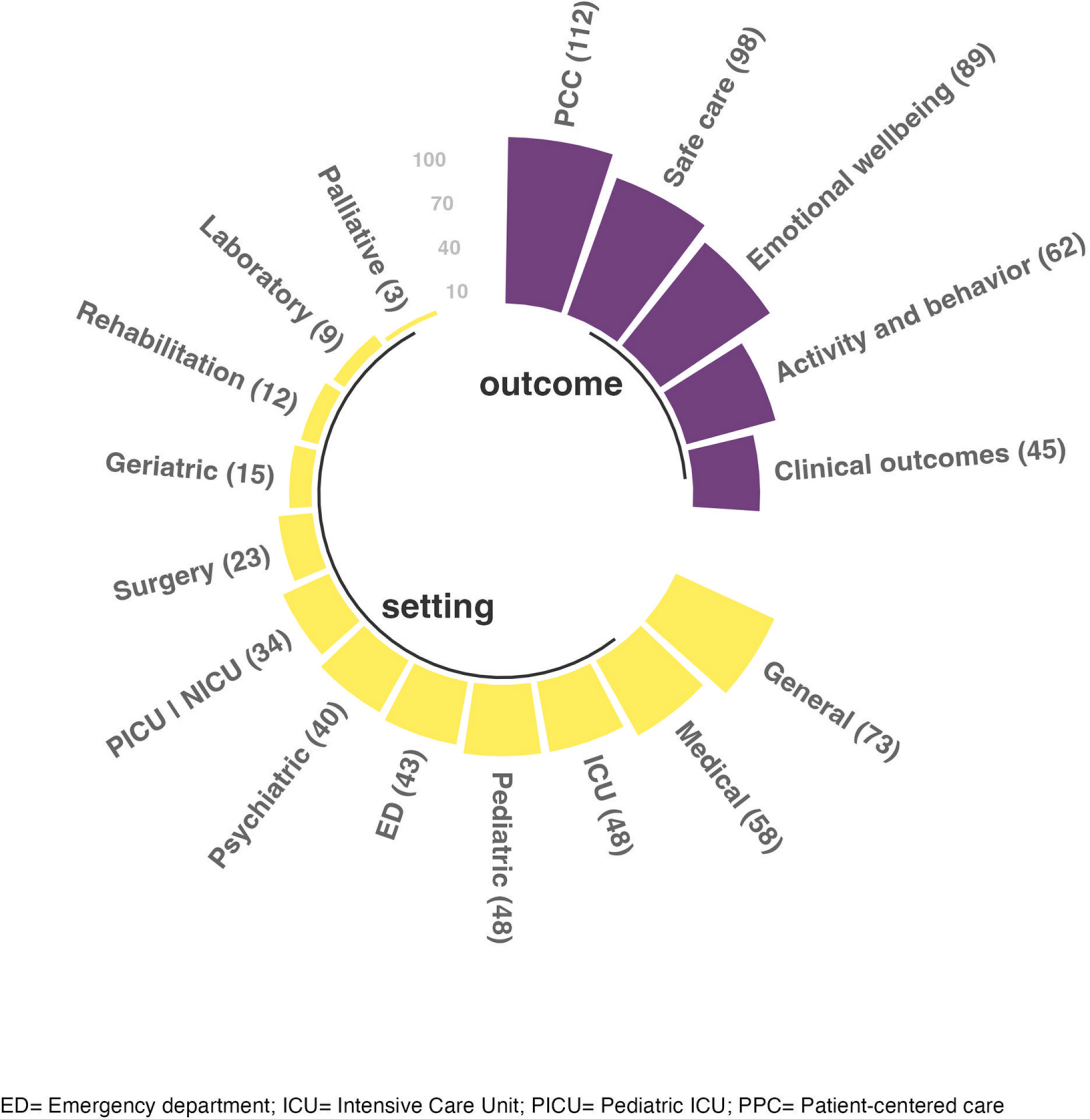
Outcomes and setting.

The settings included general wards (*n* = 73, 18%), medical wards (*n* = 58, 14%), intensive care units (ICU; *n* = 48, 12%), emergency department (*n* = 43, 11%), psychiatry (*n* = 40, 10%), pediatric (*n* = 39, 10%), surgery (*n* = 22, 5%), geriatric (*n* = 15, 4%), or rehabilitation (*n* = 12, 3%), laboratory (*n* = 9, 2%), maternal (*n* = 9, 2%), and palliative care (*n* = 3, 1%). Pediatric/neonatal ICUs; *n* = 34, 8%) and operating rooms (*n* = 1, 0.2%) were also included (Figure 4).

### Design Features of the Built Environment

Architectural features were the most studied, and 302 articles (74%) addressed patient room design, ward layout, room size, nursing stations, and/or windows for outdoor viewing and daylight (Table 2). Of these articles, 48 (16%) focused only on architectural features. The remaining 254 (84% of 302) combined architectural features with other design features, most often interior (*n* = 179, 59%) and/or social (*n* = 156, 52%) features.

**Table 2.** Design Features in included articles.

Design Feature	Number of Articles, *n* (%)
Ambient	207 (51)
Architectural	297 (73)
Interior	228 (56)
Social	180 (44)
Nature	96 (24)

*Note.* The total number of articles listed in this table is greater than the number of articles in this review because many articles reported more than one architectural feature.

Interior features were described in 229 articles (56%), which focused on artwork placement, furniture and equipment types, and wall or floor finishing. Few of these articles (*n* = 26, 11%) focused solely on interior design features.

Ambient features were described in 209 articles (51%), which addressed factors, such as lighting (natural or artificial), acoustics (noise, sounds, music, etc.), temperature, and ventilation. These were typically combined with architectural (*n* = 157, 75%), interior (*n* = 136, 65%), and/or social (*n* = 105, 5%) features. In total, 32 (15%) articles focused solely on ambient design features.

Social features were described in 180 articles (45%), which addressed privacy, control, crowding, territoriality, and communication. Ten (6%) focused on social features alone, whereas the remaining 170 (94%) examined social features in conjunction with other environmental variables.

Finally, 96 articles (24%) studied nature-related features, which included views of nature, plants, flowers, nature-inspired art, and access to nature outdoors. Of these, 80 (83%) combined natural and architectural features. Only eight articles (9%) focused exclusively on nature. Most articles (*n* = 282, 69%) examined multiple features of the built environment. Among these, 101 (25%) focused on two features, 76 (19%) on three, and 62 (15%) on four; 43 articles (11%) examined all the features.

Of the 120 articles that studied a single feature, 45 (37.5%) focused on architectural features, 31 (26%) on ambient features, 26 (22%) on interior, 10 (8%) on social, and eight (9%) focused on nature-related features.

### Studied Outcomes

Many studies (*n* = 112, 28%) investigated PCC as the primary outcome in healthcare settings, while others examined safe care (*n* = 98, 24%), emotional well-being (*n* = 89, 22%), activity and behavior (*n* = 62, 15%), and clinical outcomes (*n* = 45, 11%; Figure 4). Patients were the most common participants for all primary outcomes, except activity and behavior, which was mainly investigated in the staff (Table 3).

**Table 3.** Types of Participants and Primary Outcomes.

	Participant Types																	
Primary Outcome	Patients Only		Staff Only		Visitors Only		Patients + Staff		Patients + Visitors		Staff + Visitors		Patients + Staff + Visitors		None		Total Number of Articles	
	*n*	%	*n*	%	*n*	%	*n*	%	*n*	%	*n*	%	*n*	%	*n*	%	*n*	%
Patient-centered care	51	45.5	27	24.1	2	1.8	15	13.4	8	7.1	3	2.7	6	5.4	0	0	112	100
Safe care	56	56.6	31	31.3	0	0	8	8.1	2	2.0	1	1.0	1	1.0	0	0	99	100
Emotional well-being	38	42.7	28	31.5	2	2.3	7	7.9	3	3.4	4	4.5	7	7.9	0	0	89	100
Activity and behavior	18	29.0	25	40.3	1	1.6	11	17.7	1	1.6	1	1.6	5	8.1	0	0	62	100
Clinical outcomes	25	55.6	9	20.0	3	6.7	3	6.7	1	2.2	1	2.2	2	4.4	1	2.2	45	100
Total number of articles	188	46.2	120	29.5	8	2.0	44	10.8	15	3.7	10	2.5	21	5.2	1	0.3	407	100

*Note. n* = number of articles.

#### Person-centered care

The 112 PCC studies explored various factors related to PCC, and the majority focused on patient perspective (*n* = 51, 46%). Some also studied the experiences of staff (*n* = 27, 24%), visitors (*n* = 2, 2%), or a combination. Studied aspects include privacy, comfort, and relationships/interactions between professionals and patients. Additionally, creating an inclusive environment and personalized care were essential components.

**Figure 5.**
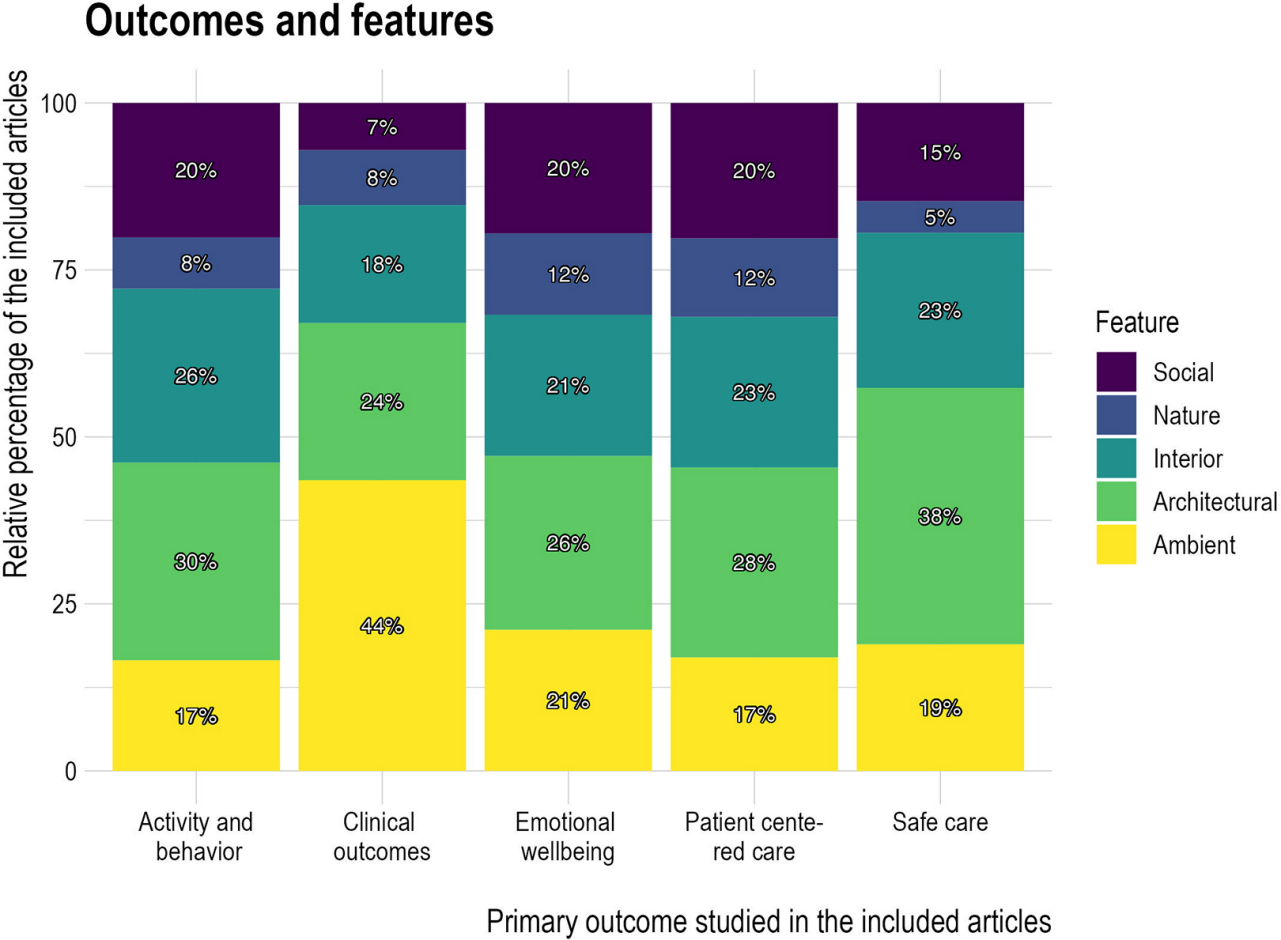
Design Features and Outcomes.

Figure 5 illustrates the categories of the design features in the studies that investigated different outcomes. Among articles that investigated activity and behavior, emotional well-being, PCC, or safe care as their primary outcome, the most investigated design feature was architectural features, followed by interior, ambient, social, and nature features. However, this pattern differed for articles that focused on clinical outcomes, with the impact of ambient features—mainly acoustic environments, such as noise and sound, lighting, such as daylight and artificial lighting, and smell—being the most investigated and nature features the least commonly investigated.

### Research Design and Methods in the Included Articles

**Figure 6.**
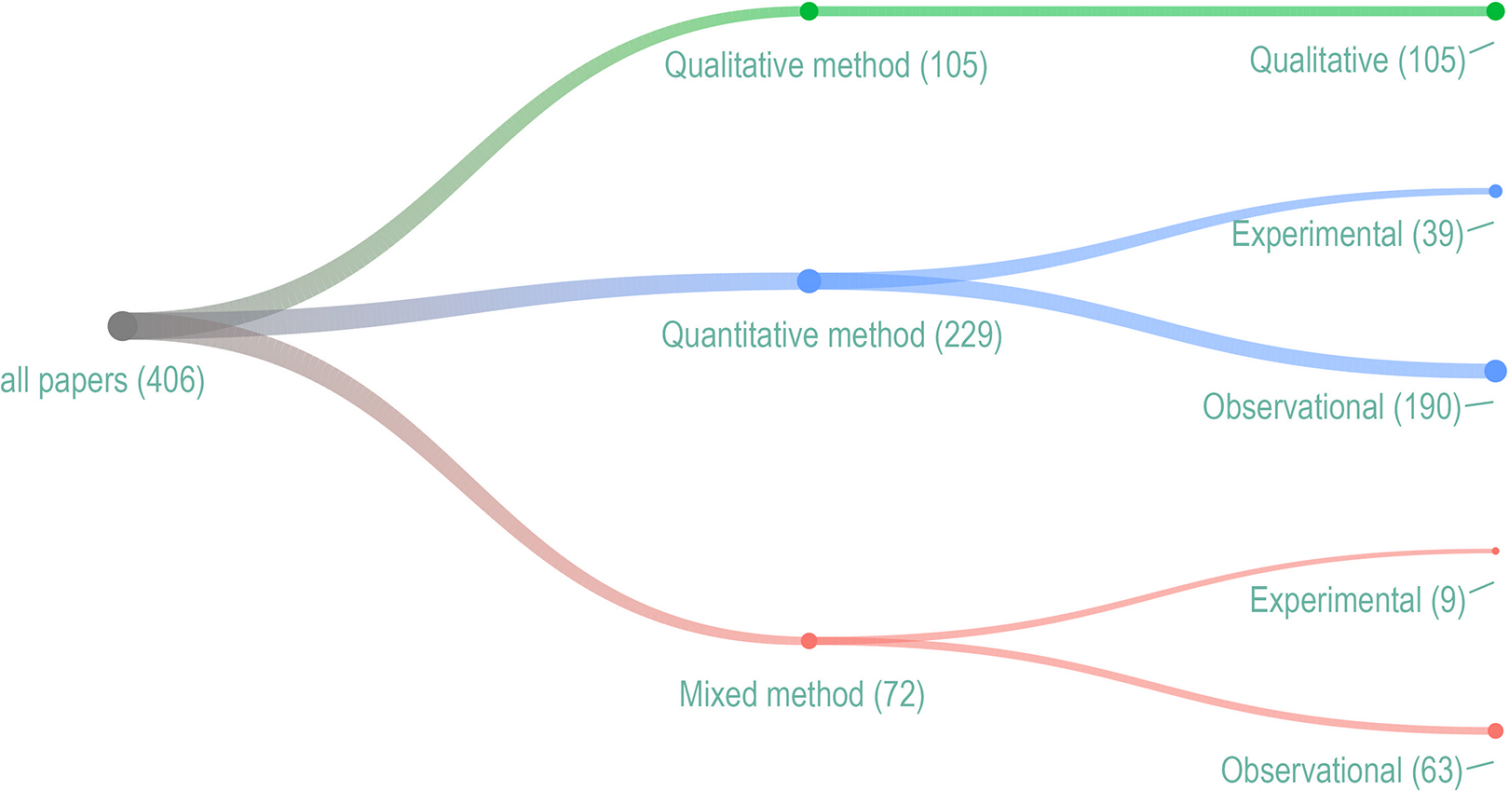
Research methods and design.

Most included articles used quantitative methods (*n* = 229, 57%; Figure 6). Of these, 39 (17%) reported experimental designs, such as randomized-controlled trials or case-crossover studies. Qualitative methods, such as observations, interviews, focus groups, photo voices, or ethnographic methods, were described in 105 studies (26%). The remaining reported studies used mixed methods (*n* = 72, 17%), some with experimental components (*n* = 9, 12.5%), and most were observational (*n* = 60, 87.5%). Limited studies (*n* = 102, 25%) used an explicit theoretical framework.

#### Methods employed depending on outcome of interest

**Figure 7.**
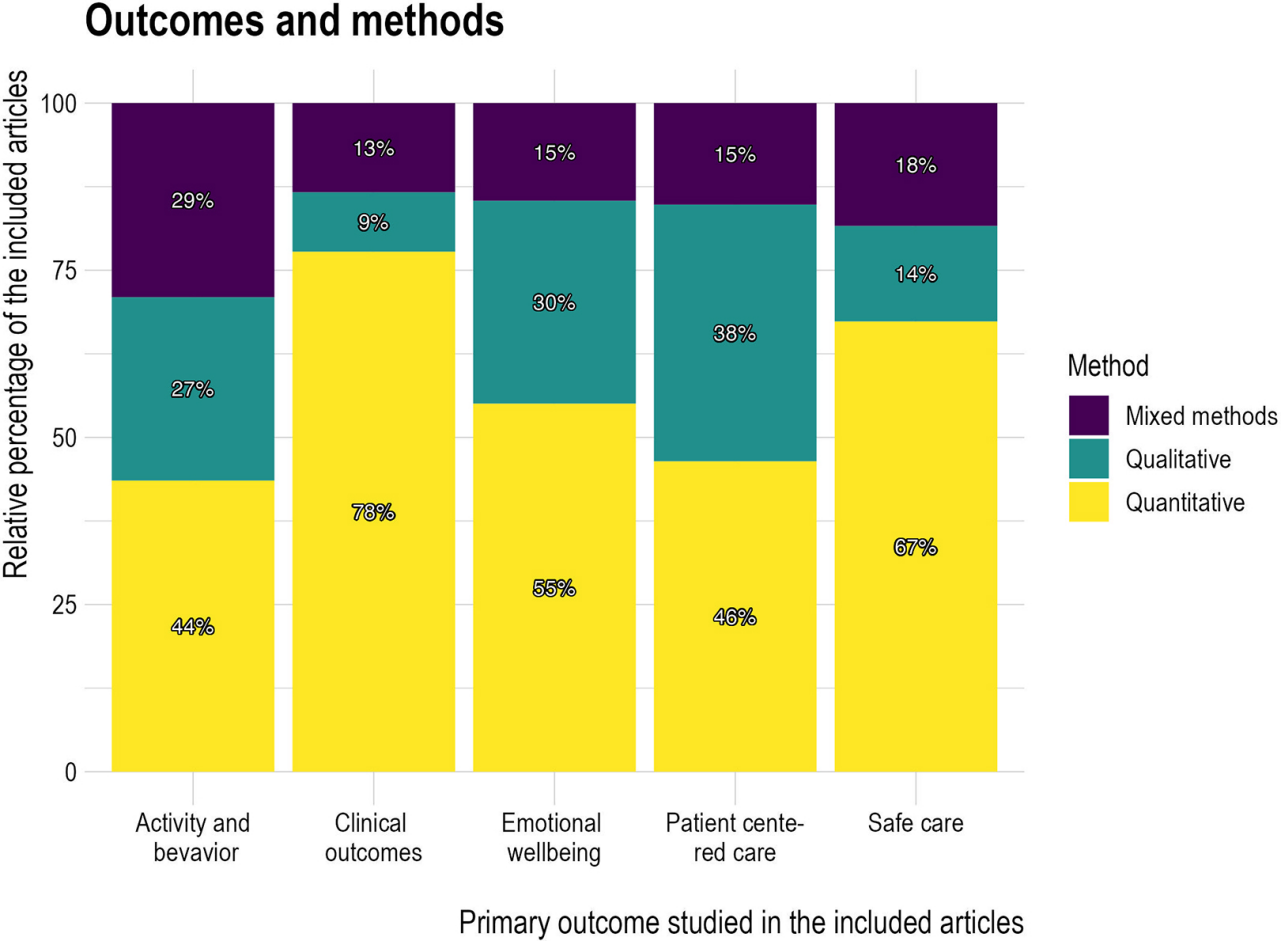
Methods and outcomes.

The methods and designs used varied based on the outcomes of interest. Quantitative methods were especially favored in articles that addressed clinical outcomes and safe care (Figure 7). Many studies used standardized assessment tools. Mixed methods were more prevalent in articles that investigated activity and behavior than in those that focused on other outcome categories. Qualitative methods were more prevalent in articles that investigated PCC than in articles that focused on other outcome categories.

#### Methods employed depending on design feature of interest

**Figure 8.**
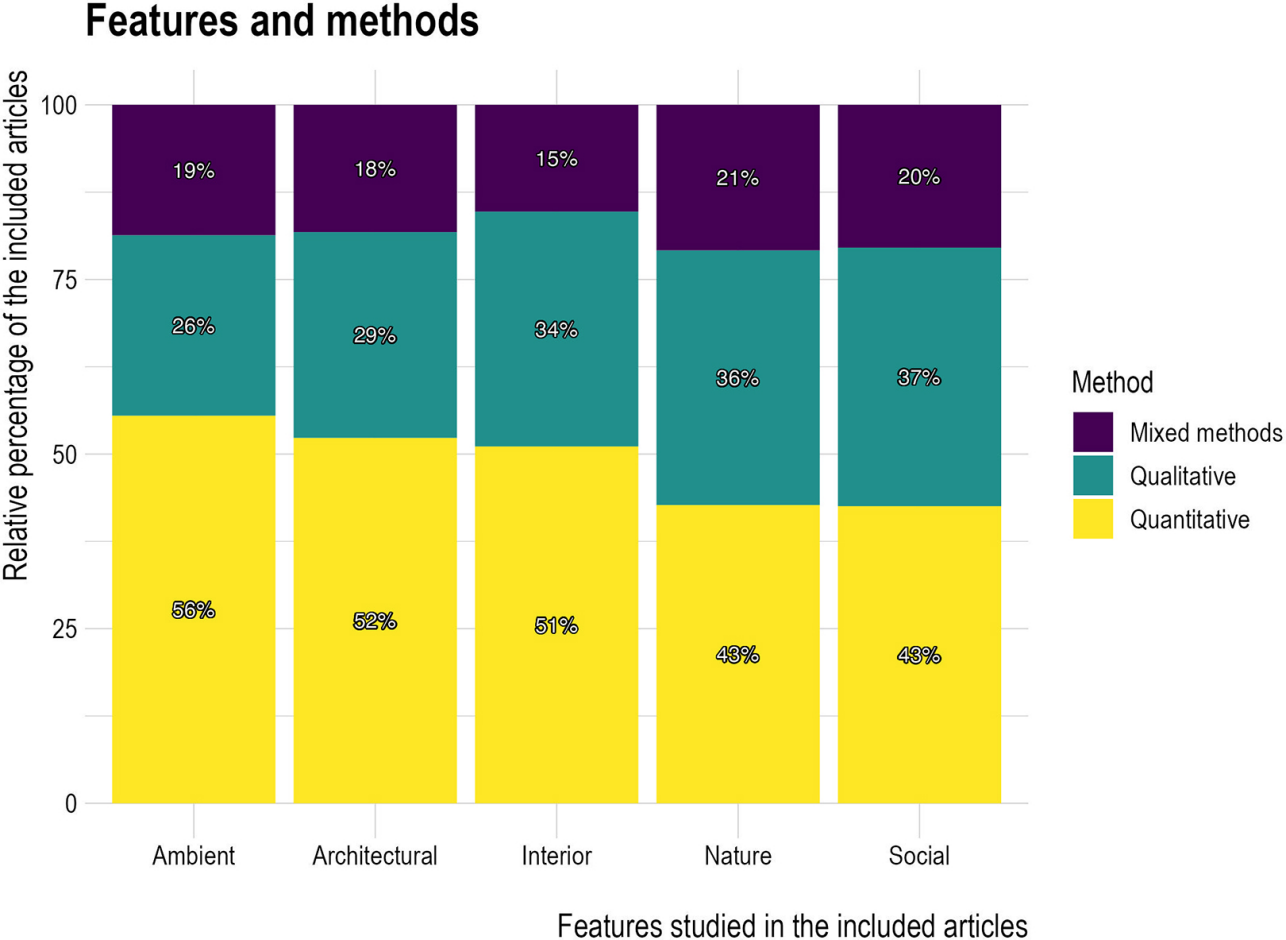
Features and methods.

The type of method used varied somewhat depending on the main environmental/design features of interest (Figure 8), with quantitative methods slightly more favored and qualitative methods slightly less favored, in studies that focused on ambient features, compared to those that focused on nature or social features.

## Discussion

The approach of the present review was distinct from the approach taken in Ulrich et al.’s (2008) review, which provided explicit design recommendations through an integrative analysis, including a segment that discussed the impact of hospital design on reducing the incidence of hospital-acquired infections. However, the aim of this study was different. Rather than focusing on specific architectural layouts like single-patient rooms, this study was anchored on well-established healthcare quality indicators.

***The approach of the present review was distinct from the approach taken in Ulrich et al.’s (2008) review, which provided explicit design recommendations through an integrative analysis, including a segment that discussed the impact of hospital design on reducing the incidence of hospital-acquired infections. However, the aim of this study was different. Rather than focusing on specific architectural layouts like single-patient rooms, this study was anchored on well-established healthcare quality indicators.***

We emphasized their importance, demonstrated how EBD was relevant in both architectural and modern healthcare practices to meet the needs of the patients and promoted the overall care quality, particularly in emerging paradigms, such as PCC, where the environment was essential (Kelly et al., 2022; McCormack & McCance, 2006).

The research on the connection between the design of the built environment and the well-being of patients and healthcare staff has increased significantly. However, there is a necessary imbalance in the distribution of research methods. There are logistical realities and challenges in conducting physical environment research in healthcare settings, such as difficulties isolating and controlling variables, resource constraints, ethical considerations, and other practical aspects. While these challenges are common to most healthcare environments research, they are particularly apparent in studies concerning outcomes that, by their very nature, are subjective and difficult to measure, such as PCC and emotional well-being. This is not to say that qualitative methods cannot yield practical, evidence-based recommendations, but rather, that all healthcare environments research—whether qualitative or quantitative—will benefit as the field matures to include more standardized practice, including more widely accepted definitions for concepts, such as PCC and emotional well-being, and standard measurement tools and theoretical frameworks for the same (van Dongen, 2022).

Our findings provided several new insights. To our best knowledge, this was the first review in which a large international and interdisciplinary team integrated the perspectives of both architecture and healthcare in the context of EBD. This unique collaboration enabled us to examine the built environment through a holistic and nuanced lens and yielding insights beyond conventional design-centric considerations. Shifting focus from individual design elements to a broader consideration of quality indicators in healthcare provided a richer understanding of the environment’s role. Hence, this review updated the existing literature and also challenged the field by reconsidering methodologies and priorities.

Limited studies explored social design features and the impact of nature. Moreover, the methodological rigor of some was questionable or uncertain due to weak methodological descriptions. Moreover, only few were based on a theoretical framework.

### Design Features

The most common design features assessed were architectural (*n* = 302, 74%), usually combined with other types of design features (interior, social, ambient, etc.). This perhaps reflected that architectural design features (ward layout, window size, etc.) were interrelated with other design features. Articles that considered a natural view from a window were classified as architectural (window size and placement) and natural design features. Half of the articles considered ambient features (lighting, noise, etc.). This may be as they were comparatively easy to quantify and measure or may reflect the growing evidence of their impact on clinical outcomes. Lighting and sound affected stress levels in patients (Münzel et al., 2022). Research that focused on ambient features and clinical outcomes emerged as a distinct category of health design research. Architectural features were the most popular design features in studies that examined PCC, safe care, emotional well-being, activity, and behavior. Studies that focused on clinical outcomes were more likely to consider ambient features as design features. Studies that focused on ambient features and clinical outcomes were also most likely to use quantitative methods. Health design research focused on ambient features and clinical outcomes will increase as more emphasis is placed on quantitative and objective methods.

This review did not explore multisensory, augmented, or immersive architectural environments, increasingly being leveraged to alleviate psychological stress and enhance the mood of patients and staff (Putrino et al., 2020). Such interventions, which included modification of the environment through projected images, scents, and sounds, offered cost-effective ways to adapt to the healthcare environment and enhance patients’ experience (Fenko & Loock, 2014; Hauck et al., 2008). Although these aspects fell outside the scope of our review, their growing importance in the healthcare context should be examined.

### Outcomes Studied

Our review revealed abundant research that investigated various aspects of PCC, which included privacy, comfort, and the relationship between healthcare professionals and patients. These studies underscored the significance of supporting an inclusive atmosphere and customizing the environment to foster PCC (McCormack & McCance, 2006). Ulrich’s (2008) review did not explicitly incorporate the concept of PCC, which pointed to shifting trends and focus within healthcare. Recently, this shift toward PCC mirrored a dynamic healthcare landscape, in which providers were increasingly expected to tailor services and care to individual patients’ needs and preferences (Ekman et al., 2011). However, research findings need to be translated into further precise guidelines for practical principles and policy measures. This translation was crucial to bridge the gap between research and practice and ensure that insights from the built environment’s role in PCC can be effectively leveraged to enhance healthcare outcomes. The healthcare system has undergone significant changes due to the rise of virtual care, earlier patient discharge, staff shortages, pandemics, and the environmental impact of healthcare facilities. These changes inevitably affect the built environment and pose challenges and opportunities for future healthcare design. Therefore, using evidence-based approaches to determine design solutions is important.

By embracing EBD principles, the healthcare and architecture fields can navigate the changes and ensure that the built environment aligns with the evolving healthcare needs. This approach enables the creation of healthcare environments that optimize patient outcomes, support the well-being of healthcare staff, and contribute to a sustainable and resilient system.

Future reviews should explore other emerging health-related topics, such as prevention and health promotion, or extend their focus to include other population groups, such as people cared for and rehabilitated in community-based settings or at home. This broadened scope can equip researchers and practitioners to address the changing needs in healthcare.

### Methods Used

Our review provided interesting insights into the research methods employed in studies that examined the relationship between built environments and various outcome measures. Studies centered on an individual’s activity and behavior as outcome measures exhibited a higher likelihood of utilizing mixed methods than those that focused on other outcome measures. Activity and behavior were often measured via researcher’s observation, which could produce both qualitative and quantitative data and benefit from a mixed methods approach (Lipson-Smith & McLaughlan, 2022). However, quantitative methods are increasingly used to address this topic as movement sensors, location tracking, and other associated technologies become more commonplace, which may provide an increased opportunity to quantitatively measure behavior without direct researcher observation.

Notably, studies that focused on natural and social features predominantly employed qualitative methods, with many examples of rigorous, rich qualitative research in this category. This less frequent use of quantitative approaches may reflect the complex and multidimensional nature of natural and social features, often not easily reducible to standardized metrics. Current quantitative tools may not sufficiently or appropriately measure these environmental aspects. This observation aligned with the findings of previous reviews (Lipson-Smith et al., 2021), which emphasized the need for more standardized yet flexible tools to quantitatively assess features within built environments and complement qualitative methods.

This gap in methodology indicated an important area for future development. Addressing this issue could involve the creation of new instruments designed specifically to measure natural and social features or adapting the existing tools to capture these elements better. A more robust quantitative understanding of these factors was required to complement the rich qualitative insights. This would allow for a more holistic understanding of the impact of built environments on health-related outcomes and enable the development of more EBD strategies.

Despite various measurement tools that accurately assessed the relationship between PCC and the built environment, their use remained limited. Standardized questionnaires designed to determine PCC could offer systematic assessments of privacy, comfort, and the relationship between healthcare professionals and patients (Edvardsson & Innes, 2010; Stewart et al., 2022). By generating standardized data, these instruments could significantly enhance our understanding of how built environments influence PCC outcomes. Further, their use facilitated more robust cross-study comparisons and developed a more cohesive, evidence-based body of literature.

Notably, the focus on single rooms diminished recently. However, the underlying question regarding the impact of room configuration on healthcare outcomes remained unclear. Research yielded compelling evidence that multiple bedrooms contributed to healthcare inequity. This was primarily owing to the disproportionate assignment of lower income patients and racial/ethnic minorities to multiple bedrooms, which significantly increased the risk of central line infections and other associated harm. The detrimental effects of multiple bedrooms extended beyond ICUs and affected patients in low-acuity general medical wards (O’Neill et al., 2018). This study highlighted the disparities in access to healthcare facilities across various countries. High-income individuals could afford private hospitals or possess upper tier insurance to secure single rooms, whereas lower income groups were disproportionately relegated to multiple bedrooms. Consequently, this disparity may have contributed significantly to the worse health outcomes.

#### Theories and definitions

Our review identified a notable gap in the utilization and specification of theoretical frameworks. Researchers must incorporate theoretical frameworks relevant to PCC to provide a solid foundation. However, there were inconsistencies. Several frameworks directly related to PCC and offered distinct interpretations and implications. The absence of a standardized approach for incorporating these frameworks contributed to the interchangeable use of central concepts, such as person-centeredness and patient participation, with other terms and concepts. This lack of clarity and consistency may lead to misunderstandings and hinder effective communication and collaboration. To advance the understanding of PCC and facilitate meaningful research, studies should explicitly utilize or specify the theoretical framework they adopt. This practice will enhance the rigor and coherence of research and promote discussions and interpretations of the findings. By establishing a common language and framework, we can foster cohesion and progress in exploring PCC and its implications in healthcare environments.

### Design Features

We observed a trend where architectural features were the most frequently examined design elements, followed by interior and social aspects. However, when the focus shifted to clinical outcomes, a significant proportion of studies delved into the impact of ambient features. We also noticed a lesser emphasis on social features in studies concerning clinical outcomes as compared to other outcomes.

The social environment, particularly in care settings for older adults, has been increasingly recognized in the recent literature (Sturge et al., 2021). This implied the need for greater attention on how social design aspects were effectively addressed to enhance clinical outcomes. The influence of social interactions on patient and staff health highlighted the need for more comprehensive investigations.

The dominance of architectural features can be attributed to their inherent tangibility, which made them more straightforward to investigate than social features. Architectural elements, such as physical layouts, offered concrete and quantifiable aspects. Conversely, social features, which encompassed elements, such as social dynamics, interpersonal relationships, and communication patterns, were intangible, complex, and context-dependent, which made them more challenging to measure and understand comprehensively.

This review revealed a notable scarcity of studies that explicitly explored the influence of social features on clinical outcomes. The articles primarily focused on social features related to privacy and social spaces and emphasized their impact on clinical and organizational benefits. However, broader social benefits, such as intentional positive social interaction and the well-being of patients and staff, should be noted. In addition, environmental features, such as air quality, noise levels, and natural distractions, had a direct impact on patient and clinical outcomes.

Architectural features went beyond aesthetics and layout; they also significantly impacted social support in healthcare settings. Noise control, which affected social support, could be influenced by both architectural and organizational factors. While organizational practices, such as keeping room doors open, exposed patients to noise, research consistently showed that architectural features, such as sound insulation, single rooms, and sound-absorbing surfaces, played a far more significant role in mitigating noise-related issues. Understanding their influence on social dynamics and clinical outcomes is crucial for designing person-centered healthcare environments. Future research should explore the integration of social features that foster positive social interactions, enhance patients’ well-being, and create supportive care cultures. Interdisciplinary collaborations between architects, healthcare professionals, and researchers are essential to ensure that architectural designs effectively meet the needs of patients, staff, and the broader healthcare community.

Understanding of the relationship between the built environment and patient safety has led to a significant focus on architectural features in healthcare buildings, as reflected in studies that investigated patients’ fall risks (Taylor et al., 2016, 2018). However, the research scope should be broadened to include and understand the role and potential of social features in enhancing the well-being of patients and staff.

Most studies (*n* = 281, 69%) described more than one environmental feature, which made their findings difficult to interpret or failed to represent the actual spatial context in a way that could be applied by architects or design practitioners. In addition, it was challenging to make cross-case comparisons when the outcomes and design feature variables were not well documented. Therefore, future research should strive to provide detailed descriptions to enable comparisons between healthcare facilities and between studies.

***Most studies (*n *= 281, 69%) described more than one environmental feature, which made their findings difficult to interpret or failed to represent the actual spatial context in a way that could be applied by architects or design practitioners. In addition, it was challenging to make cross-case comparisons when the outcomes and design feature variables were not well documented. Therefore, future research should strive to provide detailed descriptions to enable comparisons between healthcare facilities and between studies.***

Social design features, for example, were most likely to be investigated along with other design features, such as lighting or noise. This may be due to the complex interplay between social and physical environmental factors in healthcare settings, making social features more difficult to isolate and study. In addition, social features, such as communication and relationships between patients and healthcare professionals, are highly context-dependent. They could be influenced by factors beyond the built environment, such as staff training and management practices, highlighting the need for comprehensive and multifaceted studies. The result also shed light over the real complexity of healthcare and healthcare design. Thus, studying some features in isolation rather than in context could potentially bias the results.

Moreover, this review identified a mix of single- and combination-feature articles. While single-feature articles provided a focused analysis, combined feature articles provided more context for different variables in healthcare environments. This suggested that study focus had benefits, and that future research should consider the appropriate balance between focused and comprehensive analyses.

This review revealed that many studies explored natural design features also considered architectural elements. This intersection was predictable, given that visual access to natural features within and outside the built environment relied on architectural decisions, such as placing and designing openings, such as doors and windows. However, this pattern suggested that there may be less emphasis on interior greenery. This could be linked to concerns regarding possible contamination from indoor plants. Our review did not differentiate between views of or access to indoor and outdoor nature, which could benefit from further exploration.

Our findings highlighted the need for more rigorous definitions. Specifically, researchers should distinguish between different types of access to nature and their implications. This would aid in developing a more nuanced understanding of how nature, architecture, and interior design intersected and their collective impact on health-related outcomes. We expect to expand on these themes in future studies and reviews.

Despite growing research, the following question persisted: How could policymakers and designers effectively leverage these findings? Literature described a disconnection between design decisions and patient or staff outcomes, which pointed to an underutilization of research in practice (Lawson, 2013). While EBD increasingly enhanced decisions regarding purpose-built and renovated healthcare environments, more rigorous postoccupancy evaluation criteria to ensure that these environments function as intended are required (Simonsen et al., 2022).

Creating effective built environments should be perceived as a shared responsibility between healthcare providers and those planning and designing these spaces to improve care quality (Elf et al., 2015). Further research is necessary to ensure that the built healthcare environment can adapt to future healthcare needs and technological advancements. Future research should explore hospital design strategies that enable the environment to be reconfigured to changes in healthcare delivery (Pilosof, 2021; Sturge & Starrenburg, 2022).

As evidenced by the COVID-19 pandemic, healthcare infrastructures must adapt rapidly and efficiently to unforeseen circumstances and evolving needs (Sturge & Starrenburg, 2022). This readiness underscored the imperative for robust, evidence-based design and planning in response to current and future healthcare challenges.

### Strength and Limitations

The interdisciplinary nature of our team was a significant strength, enabling a comprehensive and multifaceted assessment of the data. This approach allowed for a broader perspective and deeper understanding of the complex interactions within the built environment and healthcare settings. The massive data collection effort, grounded in health indicators, provided a robust foundation for our analysis. This extensive dataset enabled a thorough exploration of the subject matter. To mitigate search bias, our review incorporated a wide range of databases, sources, journals, and MeSH terms. This diverse search strategy ensured a more inclusive and comprehensive literature capture.

However, our review encountered limitations and potential biases due to the volume of papers to be read and categorized, as well as the need for more clarity in research design, methods, and objectives in many of the included studies. It is likely that some relevant papers were missed either during the title or abstract screening process as we followed our prespecified procedures. We recommend careful consideration of resource capabilities in managing large volumes of literature and clear and detailed reporting in primary research studies. Additionally, we suggest reconstructing the framework used to categorize the design features to more distinctly separate physical design features from the experiential or social qualities they influence to provide a more nuanced analysis of the built environment. Finally, future studies should expand their scope to encompass different healthcare settings, for example, outpatient setting for a more comprehensive understanding of the built environment’s role in healthcare. Another limitation was the geographical distribution. The articles predominantly originated in Western countries, particularly North America and Scandinavian countries. This raised questions regarding whether the findings could be generalized to other cultural and geographic contexts.

This review only included studies written in English; therefore, relevant findings from other languages were not considered. These limitations highlighted the need for caution when interpreting these results and the importance of considering these factors in future research. Despite these limitations, it is important to note that we intended to create a living database continuously updated with relevant new evidence as it becomes available. Our ambition is a freely available online database that will support future researchers in the field.

## Conclusions

Through a comprehensive literature review on built environments, 406 articles that reported various environmental aspects and their influence on patients and staff were identified. However, most studies required an analytical lens and primarily presented descriptive findings. While many articles focused on the prevention or management of infections in healthcare settings, particularly in outbreak management and testing strategies, our review revealed a substantial evidence gap regarding atmosphere, art, and nature, despite emerging evidence that pointed to its crucial role in patient outcomes.

Most identified articles were descriptive case studies that offered valuable insights into specific contexts. Nevertheless, further rigorous, robust, and evaluative studies were required to inform future planning and design of healthcare facilities.

Our review emphasized the importance of more analytical studies that focused on PCC and the impact of the built environment on patient outcomes. Such studies will contribute to a better understanding of effective strategies and interventions and facilitate evidence-based decision-making by researchers and policymakers.

## Implications for Practice

- The need for a balanced focus on all aspects of the built environment, including architectural, interior, ambient, social, and nature-related features, should be emphasized in future research and practice for more comprehensive and sustainable healthcare facility design.
- Given the complexity of this research field, diverse and appropriate research methodologies tailored to the specific study outcomes and features are critical. Using quantitative, qualitative, and mixed research methods can provide a comprehensive and nuanced understanding, enhancing the quality of healthcare provision.
- Insights from focus areas such as PCC and safe care should be leveraged to inform design interventions. For instance, designing spaces that foster better interaction between patients and healthcare providers could be an area for further exploration based on our analysis.
- Future research and policy development should also align with sustainability objectives guided by the United Nations Sustainable Development Goals. These insights will contribute significantly toward creating healthcare facilities that are efficient, effective, sustainable, and adaptable to climate change impacts.
- Our study underlines the crucial role of open-access databases in accelerating empirical research by allowing quick access to relevant studies. For instance, an open database could help practitioners locate evidence-based design strategies for enhancing patient satisfaction or safety in inpatient healthcare settings.

## Supplemental Material

Supplemental Material, sj-pdf-1-her-10.1177_19375867241251830 - A Systematic Review of Research Gaps in the Built Environment of Inpatient Healthcare Settings Supplemental Material, sj-pdf-1-her-10.1177_19375867241251830 for A Systematic Review of Research Gaps in the Built Environment of Inpatient Healthcare Settings by Marie Elf, Ruby Lipson-Smith, Maya Kylén, Juan Pablo Saa, Jodi Sturge, Elke Miedema, Susanna Nordin, Julie Bernhardt and Anna Anåker in HERD: Health Environments Research & Design Journal

Supplemental Material, sj-pdf-2-her-10.1177_19375867241251830 - A Systematic Review of Research Gaps in the Built Environment of Inpatient Healthcare Settings Supplemental Material, sj-pdf-2-her-10.1177_19375867241251830 for A Systematic Review of Research Gaps in the Built Environment of Inpatient Healthcare Settings by Marie Elf, Ruby Lipson-Smith, Maya Kylén, Juan Pablo Saa, Jodi Sturge, Elke Miedema, Susanna Nordin, Julie Bernhardt and Anna Anåker in HERD: Health Environments Research & Design Journal
