# Supplementary material for: A Systematic Review of Research Gaps in the Built Environment of Inpatient Healthcare Settings
Source: HERD. 2024 May 28;17(3):372–94. doi: 10.1177/19375867241251830 (PMC11491052; doi:10.1177/19375867241251830)
Supplement: Supplemental Material, sj-pdf-1-her-10.1177_19375867241251830 - A Systematic Review of Research Gaps in the Built Environment of Inpatient Healthcare Settings [file sj-pdf-1-her-10.1177_19375867241251830.pdf]

Supplementary file 1. Boolean search terms by main topics

Architecture	Patient(s)	Healthcare setting(s)
AND	*AND*	
OR	*OR*	*OR*
Architectural design	Client(s)	Healthcare facility(ies)
Building design	Consumer(s)	Healthcare space(s)
Environment design,	Family	Healthcare building(s)
Physical environment	Relatives	Health facility (ies)
Built environment Health	Visitor(s)	Hospital(s)
Health Facility Environment	Caregiver(s)	Healthcare service(s)
Evidence-based Facility Design	Health Personnel	E-health
Evidence-based Design	Staff	Telemedicine
Environmental design	Nurse(s)	Telehealth
Hospital construction	Physician(s)	Medical home(s)
Hospital Design and Construction		Patient-centered medical home
Facility design		Ambulatory Care Facility(ies)
Universal design		Ward(s)
Interior design		Emergency department(s)
Garden(s)		Intensive care
		Critical care
		Acute care environment(s)
		Care unit(s)
		Inpatient
		Waiting room
		Accident and emergency
		NICU
		Rehabilitation room
		PICU
		Operating room
