# Supplementary material for: A Systematic Review of Research Gaps in the Built Environment of Inpatient Healthcare Settings
Source: HERD. 2024 May 28;17(3):372–94. doi: 10.1177/19375867241251830 (PMC11491052; doi:10.1177/19375867241251830)
Supplement: Supplemental Material, sj-pdf-2-her-10.1177_19375867241251830 - A Systematic Review of Research Gaps in the Built Environment of Inpatient Healthcare Settings [file sj-pdf-2-her-10.1177_19375867241251830.pdf]

Supplementary file 2. Data extraction file

nr	Author	Year	Settings	Qualitative	Quantitative	Mixed methods	Research Design	Activity & behaviour	Clinical outcomes	Emotional wellbeing	PCC	Safe care	Quality	Ambient	Architectural	Interrior	Social	Nature	Patients	Personell	Visitors
1	Aburas	2017	Maternal		●		Experimental		●				51%					●	●		
2	Adams	2010	Pediatric	●			Qualitative	●					52%	●	●	●	●	●	●		
3	Agrest	2018	Psychiatric	●			Qualitative			●			76%			●			●		
4	Ahamed et al.	2018	PICU/NICU		●		Observational		●				68%	●							
5	Al-Motlaq	2018	PICU/NICU			●	Observational				●		76%	●	●	●	●			●	
6	Alexiou	2016	Psychiatric		●		Observational				●		53%	●	●	●	●	●	●		
7	Alexiou et al.	2018	Psychiatric		●		Observational				●		82%				●			●	
8	Alfa & Öztürk	2019	General		●		Observational			●			72%	●	●				●		
9	Ali	2018	General		●		Observational					●	68%		●				●		
10	Alomari	2018	Pediatric			●	Observational					●	76%		●	●				●	
11	Alvaro	2016	Rehabilitation		●		Observational				●		53%		●			●	●	●	
12	Amankwah et al.	2019	General		●		Observational				●		82%				●		●		
13	Anaker	2017	Medical			●	Observational	●					54%	●	●	●	●		●		
14	Anaker	2018	Medical			●	Observational	●					76%	●	●	●	●		●		
15	Anåker	2018	Medical	●			Qualitative				●		71%				●	●	●		
17	Andrade	2017	Surgery		●		Observational					●	57%	●	●	●	●		●		
18	Annemans	2016	Surgery	●			Qualitative				●		81%		●	●	●		●		
19	Annemans	2018	ED	●			Qualitative				●		71%		●	●	●		●		
20	Annemans et al.	2018	General	●			Qualitative				●		86%		●	●	●	●	●		
21	Apple	2014	ICU			●	Observational			●			67%	●						●	
22	Applebaum	2010	ED		●		Observational		●				59%	●	●	●				●	
23	Arbel et al.	2019	Rehabilitation			●	Observational				●		88%	●				●	●		
24	Arenson	2013	ICU		●		Observational			●			78%	●	●	●			●		
25	Ariza-Vega et al.	2019	Geriatric		●		Observational	●					58%		●	●			●		●
26	Bail	2013	Geriatric			●	Observational					●	56%		●					●	
27	Balm	2013	ICU			●	Observational					●	55%		●				●	●	
28	Bayramzadeh	2014	Medical		●		Observational				●		77%		●		●			●	
29	Bayramzadeh	2018	Psychiatric		●		Observational					●	68%	●	●	●	●		●		
30	Bazuin	2015	PICU/NICU			●	Observational		●				53%		●	●				●	
31	Beckstrand et al.	2018	ED			●	Observational				●		76%		●		●			●	
32	Beckstrand et al.	2019	PICU/NICU			●	Observational				●		76%		●		●			●	
33	Bekhof et al.	2019	Pediatric		●		Observational		●				65%		●				●		
34	Berry & Robertson	2019	Psychiatric		●		Observational			●			83%		●					●	
35	Beukeboom	2012	Laboratory		●		Experimental		●				65%					●	●		
36	Bevan et al.	2019	Pediatric		●		Observational				●		80%	●					●		●
37	Biagioli	2016	General	●			Qualitative			●			57%		●		●		●		
38	Biddiss	2018	Pediatric		●		Observational			●			59%			●			●		
39	Birnbach	2010	Surgery		●		Experimental					●	67%		●	●				●	
40	Blandfort et al.	2020	Geriatric		●		Observational				●		77%		●		●		●		
41	Blaschke	2017	Medical		●		Observational			●			54%					●	●	●	●
42	Blaschke et al.	2018	Medical	●			Qualitative				●		81%					●	●		
43	Blennerhassett	2018	Rehabilitation		●		Observational	●					61%	●	●	●	●	●	●		
44	Bonuel	2013	General	●			Qualitative			●			86%		●		●			●	
45	Boog	2013	ICU			●	Observational	●					60%			●				●	
46	Borgmann	2014	Surgery		●		Experimental					●	79%		●	●			●		
47	Borhani	2016	ICU	●			Qualitative				●		71%	●		●	●		●	●	
48	Boylan et al.	2019	Medical		●		Observational				●		59%		●		●		●		
49	Bracken-Scally et al.	2019	ED			●	Observational		●				62%	●	●				●	●	●
50	Brewer	2018	General		●		Observational					●	69%		●		●		●		
51	Broadbent	2014	ED	●			Qualitative	●					57%	●	●	●	●			●	
52	Brooke & Semlyen	2019	Geriatric	●			Qualitative				●		58%		●	●	●		●		●
53	Broom	2018	PICU/NICU		●		Observational	●					63%		●					●	
54	Browall	2013	Medical	●			Qualitative					●	62%				●		●		
55	Brown-Johnson et al.	2019	General			●	Observational			●			59%		●					●	
56	Brown et al.	2019	General	●			Qualitative					●	64%	●	●	●	●			●	
57	Bukh	2015	Medical		●		Observational				●		52%	●		●	●		●	●	
58	Bulu	2019	General		●		Observational				●		63%				●		●		
59	Butler et al.	2019	PICU/NICU	●			Qualitative	●					57%				●				●
60	Buxton	2012	Laboratory		●		Observational		●				85%	●					●		
61	Calnan	2013	ED	●			Qualitative				●		67%		●	●			●	●	●

nr	Author	Year	Settings	Qualitative	Quantitative	Mixed methods	Research Design	Activity & behaviour	Clinical outcomes	Emotional wellbeing	PCC	Safe care	Quality	Ambient	Architectural	Interior	Social	Nature	Patients	Personell	Visitors
62	Campos Andrade	2013	Surgery		●		Observational			●			85%				●		●		
63	Camuccio et al.	2019	Psychiatric		●		Observational		●				85%	●					●		
64	Cartland	2018	Pediatric		●		Observational			●			68%		●	●	●		●		
65	Caruso	2014	ICU		●		Observational		●				64%		●	●	●		●		
66	Catt	2018	Geriatric			●	Observational			●			55%		●	●		●		●	
67	Chahal	2012	Medical		●		Observational				●		75%	●		●			●		
68	Chekol	2016	Maternal			●	Observational				●		71%	●	●				●		
69	Cheng	2010	ICU		●		Observational					●	75%	●	●	●			●		
70	Chiu	2018	ICU		●		Observational					●	90%	●	●				●		
71	Choi	2013	ICU		●		Observational				●		73%				●		●		
72	Chou et al.	2018	General		●		Observational				●		59%		●		●		●	●	
73	Chrysikou	2019	Psychiatric			●	Observational				●		69%		●				●	●	
74	Cloutier	2016	General		●		Experimental					●	88%		●	●			●		
75	Cohen et al.	2019	General		●		Observational					●	89%		●		●		●		
76	Colley	2018	Rehabilitation			●	Observational	●					62%		●				●	●	
77	Combariza	2018	Medical		●		Experimental					●	58%	●					●		
78	Cone	2010	PICU/NICU		●		Observational					●	60%	●	●	●	●			●	
79	Copeland	2017	Medical		●		Observational			●			58%		●		●		●	●	
80	Cordoza et al.	2018	General		●		Experimental			●			62%					●		●	
81	Crane	2016	General		●		Experimental	●					57%			●			●	●	
82	Crawford et al.	2018	ICU		●		Observational		●				60%	●						●	
83	Cure	2015	General		●		Observational	●					63%			●				●	
84	Curtis	2017	Pediatric	●			Qualitative				●		60%				●		●	●	●
85	Cusack et al.	2019	General			●	Observational				●		75%		●		●		●	●	
86	D souza et al.	2019	General		●		Observational		●				63%		●		●		●		
87	Daemen	2014	Medical			●	Observational		●				69%	●						●	
88	Daniels	2016	PICU/NICU		●		Observational		●				75%	●					●		
89	Davis	2011	Laboratory			●	Observational	●					81%					●	●	●	
90	Davis et al.	2019	General			●	Observational					●	75%		●		●		●	●	
91	de Korne	2012	Surgery			●	Observational	●					67%	●		●				●	
92	de Matos et al.	2019	ICU		●		Observational			●			53%		●		●			●	●
93	Degl' Innocenti et al.	2019	Psychiatric		●		Observational				●		75%	●	●	●	●	●	●		
94	Dendaas	2011	ED		●		Observational					●	57%	●	●	●	●			●	
95	Dennis	2010	Medical		●		Experimental		●				73%	●					●		
96	Devlin	2016	Surgery	●			Qualitative			●			90%	●	●	●	●	●	●		
97	Deyneko	2016	General		●		Observational					●	76%		●					●	
98	Dianat	2013	General		●		Observational		●				50%	●						●	
99	Digby	2014	Geriatric	●			Qualitative			●			67%	●	●	●	●	●	●		●
100	DiGiacomo et al.	2019	ICU		●		Observational					●	82%	●		●			●		
101	Ding	2017	ICU	●			Qualitative			●			86%	●	●				●	●	●
102	Djukic	2014	General		●		Observational				●		65%	●	●	●				●	
103	Doig	2010	Geriatric		●		Experimental			●			82%			●			●		
104	Doig	2011	General	●			Qualitative					●	62%		●				●	●	●
105	Dolan et al.	2019	General		●		Observational				●		79%		●	●	●		●	●	●
106	Domanico	2010	PICU/NICU		●		Observational				●		60%	●	●		●			●	●
107	Domanico	2011	PICU/NICU		●		Observational					●	89%	●	●		●		●		
108	Donald	2015	Psychiatric	●			Qualitative				●		71%		●		●	●	●		
109	Donetto	2017	ED	●			Qualitative			●			67%	●	●		●			●	
110	Dowling	2012	PICU/NICU		●		Observational			●			69%		●		●		●		
111	Drahota	2013	General		●		Experimental					●	86%			●			●	●	
112	DuBose et al.	2018	General	●			Qualitative					●	67%	●	●	●				●	●
113	Edgerton	2010	Psychiatric		●		Observational	●					75%	●	●	●	●	●	●		
114	Edwards & Singh	2016	Geriatric			●	Observational			●			71%	●	●		●		●		
115	Ellison	2014	Medical		●		Observational					●	79%		●				●		
116	Emami	2018	Medical		●		Experimental			●			55%		●			●	●		
117	Fay	2017	General		●		Observational	●					52%		●		●			●	
118	Feeley et al.	2019	PICU/NICU		●		Observational					●	67%	●	●		●	●		●	
119	Feeley et al.	2020	PICU/NICU		●		Observational				●		73%	●	●	●			●		●
120	Fenko	2014	Surgery		●		Experimental			●			52%	●					●		
121	Ferri	2015	ICU	●			Qualitative			●			62%	●	●	●	●	●		●	●
122	Flacking	2013	PICU/NICU	●			Qualitative				●		62%		●		●			●	●

nr	Author	Year	Settings	Qualitative	Quantitative	Mixed methods	Research Design	Activity & behaviour	Clinical outcomes	Emotional wellbeing	PCC	Safe care	Quality	Ambient	Architectural	Interrior	Social	Nature	Patients	Personell	Visitors
123	Ford	2017	Medical		●		Observational					●	85%			●			●		
124	France	2016	Pediatric			●	Observational					●	53%	●	●	●	●	●		●	
125	Frechette et al.	2020	PICU/NICU	●			Qualitative				●		86%		●	●				●	
126	García Marcos	2014	Laboratory		●		Observational				●		60%			●			●		
127	Gardiner	2011	Palliative	●			Qualitative				●		65%			●	●			●	
128	Gbyl	2016	Psychiatric		●		Experimental			●			67%	●	●				●		
129	Gesser-Edelsburg & Birma	2018	General	●			Qualitative				●		86%	●	●	●		●		●	
130	Gharaveis	2016	Rehabilitation			●	Observational	●					62%	●	●			●	●		
131	Gharaveis et al.	2018	ED			●	Observational					●	62%	●	●		●			●	
132	Gharaveis et al.	2019	ED	●			Qualitative	●					62%	●	●	●	●		●	●	
133	Gharaveis et al.	2019	ED			●	Observational					●	67%	●	●	●	●			●	
134	Gharaveis et al.	2019	ED			●	Observational					●	71%		●		●			●	
135	Giesbrecht	2018	Palliative	●			Qualitative			●			76%	●	●	●	●	●	●		
136	Gimenez	2017	Medical		●		Experimental			●			63%	●					●		
137	Goeren et al.	2018	ICU		●		Observational		●				72%	●					●	●	
138	Goto	2017	Geriatric		●		Experimental					●	52%	●	●			●	●		
139	Griepentrog et al.	2018	ICU		●		Observational		●				68%	●						●	
140	Gum	2012	ED	●			Qualitative	●					62%		●		●			●	
141	Gunn	2015	General	●			Qualitative				●		62%		●		●			●	
142	Haddox	2018	Geriatric	●			Qualitative	●					62%	●	●	●	●		●		
143	Hajradinovic et al.	2018	ED	●			Qualitative				●		72%		●	●	●	●	●		
144	Hall et al.	2019	Maternal			●	Observational	●					62%		●		●			●	
145	Ham	2015	General		●		Observational				●		58%		●	●			●		
146	Hamel	2010	Medical		●		Observational					●	78%		●				●		
147	Hammond	2014	Maternal	●			Qualitative	●					67%	●	●	●				●	
148	Hammond	2017	Maternal	●			Qualitative			●			62%	●	●	●	●			●	
149	Han et al.	2018	General		●		Observational			●			72%	●	●	●	●	●		●	
150	Hanger	2017	Geriatric		●		Observational					●	67%			●			●		
151	Hanson	2013	Medical		●		Observational			●			56%			●			●		
152	Harris	2015	Medical		●		Observational		●				57%	●		●			●	●	
153	Harte	2016	Maternal	●			Qualitative			●			76%	●	●	●	●		●	●	●
154	Hartigan et al.	2018	ED			●	Observational				●		69%		●		●	●	●		
155	Haywood	2018	Rehabilitation	●			Qualitative	●					67%		●	●	●		●	●	●
156	Henize et al.	2018	General		●		Observational				●		68%		●	●	●		●		
157	Herlihey	2017	Laboratory			●	Experimental	●					82%			●	●			●	
158	Hesselink et al.	2020	Surgery	●			Qualitative				●		76%	●	●	●	●	●	●		
159	Hignett	2010	PICU/NICU			●	Experimental	●					63%	●	●				●	●	
160	Higuera-Trujillo et al.	2020	Pediatric		●		Observational		●				62%	●							●
161	Hor	2014	ICU	●			Qualitative	●					62%		●	●	●			●	
162	Hoybye	2013	Medical	●			Qualitative				●		62%	●		●	●		●		
163	Hung	2014	Psychiatric	●			Qualitative			●			86%	●	●		●		●		●
164	Hung	2017	General	●			Qualitative					●	90%	●	●	●	●		●		
165	Hunter	2017	ED	●			Qualitative				●		67%	●	●	●	●			●	
166	Hutton	2010	General	●			Qualitative				●		76%			●			●	●	
167	Huynh et al.	2020	ICU		●		Observational	●					67%		●				●	●	●
168	Innes	2014	ED			●	Observational			●			52%	●		●	●			●	
169	Isobel	2015	Psychiatric			●	Observational				●		62%			●	●	●		●	
170	Jelinek	2013	ED	●			Qualitative			●			66%	●	●		●			●	
171	Jellema et al.	2020	Medical	●			Qualitative				●		82%	●	●	●	●	●	●	●	●
172	Jenkins	2015	Psychiatric		●		Observational	●					53%		●				●		
173	Johansson	2012	ICU			●	Observational		●				58%	●					●		
174	Johansson	2012	ICU	●			Qualitative		●				62%	●					●		
175	Johansson et al.	2018	ICU		●		Experimental		●				78%	●					●		
176	Jones	2016	PICU/NICU			●	Observational				●		67%				●		●	●	●
177	Jones	2016	Pediatric		●		Observational	●					76%	●	●				●		
178	Jongerden	2013	ICU		●		Observational				●		73%	●		●	●		●		●
179	Joshi et al.	2018	PICU/NICU		●		Observational					●	72%	●	●				●		
180	Jou	2015	General		●		Observational					●	83%		●				●		
181	Julian	2015	PICU/NICU		●		Observational					●	89%		●				●		
182	Kain	2011	PICU/NICU	●			Qualitative	●					75%	●	●	●	●			●	
183	Kalantari	2017	Psychiatric			●	Observational			●			58%		●	●	●			●	

nr	Author	Year	Settings	Qualitative	Quantitative	Mixed methods	Research Design	Activity & behaviour	Clinical outcomes	Emotional wellbeing	PCC	Safe care	Quality	Ambient	Architectural	Interrior	Social	Nature	Patients	Personell	Visitors
184	Kawai et al.	2019	PICU/NICU		●		Observational		●				62%	●					●		
185	Kelley	2011	ED	●			Qualitative	●					50%		●	●			●	●	
186	Khan	2016	Surgery			●	Experimental			●			50%	●				●	●		
187	Killington et al.	2019	Rehabilitation	●			Qualitative				●		58%		●	●	●	●	●	●	●
188	Kim	2014	Geriatric		●		Observational				●		66%	●	●	●	●	●		●	
189	Kim	2015	General		●		Observational					●	58%	●	●	●	●	●		●	
190	Kitchens	2018	General	●			Qualitative					●	81%		●	●	●		●		●
191	Kohn	2013	ICU		●		Observational					●	72%	●	●				●		
192	Koller	2014	Pediatric	●			Qualitative			●			62%	●	●	●	●	●	●		
193	Kossow	2017	Surgery		●		Observational					●	72%		●	●			●		
194	Kotzer	2011	Pediatric		●		Observational			●			50%	●	●		●			●	
195	Krugman	2015	General		●		Observational			●			88%	●	●	●	●			●	
196	Kudchadkar	2016	Pediatric		●		Observational		●				65%	●	●					●	
197	Lambert	2014	Pediatric	●			Qualitative	●					67%	●	●	●	●	●	●		
198	Lambert	2014	Pediatric	●			Qualitative			●			62%		●	●	●	●	●		
199	Larsen	2014	Medical	●			Qualitative				●		62%				●		●		
200	LaVela	2016	General		●		Observational				●		70%		●	●	●		●		
201	Lavender	2015	Medical	●			Qualitative	●					67%	●	●	●	●			●	
202	Lazar	2015	Pediatric		●		Observational					●	61%		●				●		
203	Leaf	2010	ICU		●		Observational					●	77%		●	●			●		
204	Lee	2014	General		●		Observational				●		80%		●				●		
205	Lester	2014	PICU/NICU		●		Observational			●			79%		●				●		
206	Levin	2011	ICU		●		Observational					●	79%		●				●		
207	Lin	2013	General		●		Observational					●	52%			●	●	●		●	
208	Lin et al.	2019	ICU	●			Qualitative					●	62%		●		●			●	
209	Liu	2014	Medical	●			Qualitative				●		79%		●	●			●	●	
210	Lo Verso	2016	General		●		Observational		●				73%	●					●	●	●
211	Locatelli	2015	Medical	●			Qualitative				●		62%	●	●	●		●		●	
212	Lones	2016	Medical		●		Observational					●	70%		●	●			●	●	●
213	Long	2011	Psychiatric		●		Observational			●			60%	●	●	●	●	●	●	●	
214	Lopez-Tarruella et al.	2019	General		●		Observational			●			76%	●		●					●
215	Lorenz	2011	Medical		●		Observational					●	58%		●				●		
216	Lu	2014	ICU		●		Observational				●		59%		●				●		
217	Lu	2017	Medical		●		Observational					●	54%		●		●		●		
218	Maben	2016	ED			●	Observational					●	72%	●	●	●	●	●	●	●	
219	MacAllister	2016	General			●	Observational				●		57%	●			●	●	●		
220	MacAllister	2018	Medical		●		Observational				●		55%		●				●		
221	MacKay, Peggy	2019	Surgery		●		Experimental					●	62%	●	●				●		
222	Madan	2014	Psychiatric		●		Observational				●		83%	●	●	●		●	●		
223	Maddah et al.	2019	Medical		●		Experimental		●				78%	●		●			●		
224	Magdzinski et al.	2018	ICU	●			Qualitative				●		56%	●	●		●			●	
225	Mahmood	2010	ED		●		Observational	●					60%	●	●	●			●		
226	Mahmood	2011	ED		●		Observational	●					85%	●	●	●	●			●	
227	Mahmood & Tayib (a)	2019	General		●		Observational				●		62%	●	●	●	●	●	●	●	
228	Mahmood & Tayib (b)	2019	General		●		Observational				●		58%	●	●	●	●	●	●		
229	Maloret	2018	Psychiatric	●			Qualitative			●			76%	●	●		●		●		
230	Manganelli	2013	Rehabilitation		●		Observational	●					67%			●			●	●	
231	Margolies	2015	Pediatric		●		Experimental	●					56%			●	●		●	●	●
232	Mazzei	2014	Psychiatric		●		Experimental	●					60%	●	●	●	●	●	●		
233	McCurdy	2015	Psychiatric		●		Experimental	●					67%		●				●		
234	McGough	2018	Medical		●		Observational					●	62%	●	●	●	●		●	●	
235	Minnick et al.	2019	Pediatric		●		Observational					●	59%	●	●	●				●	
236	Missildine	2010	Medical		●		Observational		●				64%	●	●				●		
237	Mogensen	2018	Medical	●			Qualitative	●					67%			●	●		●	●	
238	Mondy	2016	Maternal	●			Qualitative			●			62%	●	●	●	●	●		●	
239	Morris et al.	2019	Psychiatric		●		Observational				●		62%	●	●	●			●		
240	Motzek	2015	Geriatric		●		Experimental	●					61%		●	●			●	●	●
241	Motzek	2017	Medical		●		Experimental	●					96%			●			●		
242	Mourshed	2012	General			●	Observational				●		57%	●	●	●		●		●	
243	Munier-Marion	2016	General		●		Observational					●	62%		●				●		
244	Murphy	2010	Geriatric		●		Observational			●			75%		●			●	●		

nr	Author	Year	Settings	Qualitative	Quantitative	Mixed methods	Research Design	Activity & behaviour	Clinical outcomes	Emotional wellbeing	PCC	Safe care	Quality	Ambient	Architectural	Interior	Social	Nature	Patients	Personell	Visitors
245	Naccarella	2018	ED			●	Observational				●		56%		●					●	
246	Nanda	2011	Psychiatric			●	Experimental	●					50%	●	●		●		●	●	
247	Nanda	2015	Medical			●	Observational			●			77%			●				●	
248	Nasab et al.	2020	Pediatric	●			Qualitative				●		58%	●	●	●	●	●	●		
249	Nazarian et al.	2018	General	●			Qualitative					●	62%		●					●	
250	Nejati	2016	General			●	Observational			●			78%	●	●	●	●	●		●	
251	Nejati	2016	General		●		Observational			●			65%	●	●	●		●		●	
252	Nielsen	2017	Medical			●	Experimental			●			57%			●			●		
253	Nielsen	2018	Surgery		●		Experimental			●			58%	●		●			●		
254	Nunes	2011	Medical		●		Observational					●	61%	●					●	●	
255	Nyrud	2014	General		●		Observational			●			62%			●				●	
256	Ó Coimín et al.	2019	General		●		Observational				●		68%		●				●		
257	O'Halloran	2011	Medical	●			Qualitative				●		81%	●	●	●			●		
258	O'Reilly	2016	ICU		●		Observational				●		77%		●	●				●	
259	O'Neill	2018	General		●		Observational					●	78%		●				●		
260	Okcu	2011	ICU		●		Observational		●				94%	●	●					●	
261	Okkels et al.	2020	Psychiatric		●		Experimental		●				72%	●					●		
262	Olausson	2012	ICU	●			Qualitative				●		86%	●	●	●		●			●
263	Olausson	2013	ICU	●			Qualitative				●		62%	●	●	●	●	●	●		
264	Olausson	2014	ICU	●			Qualitative				●		77%	●	●	●		●		●	
265	Olausson et al.	2019	Psychiatric	●			Qualitative				●		68%		●	●			●		
266	Palmer	2013	OR		●		Observational	●					64%		●	●			●	●	
267	Paraskevopoulou	2018	Psychiatric		●		Experimental			●			52%					●	●		
268	Park	2013	Pediatric		●		Experimental		●				60%			●		●	●		
269	Pasha	2013	Pediatric			●	Observational			●			67%		●	●		●		●	●
270	Pati	2010	Laboratory			●	Experimental	●					94%		●					●	
271	Pati	2012	Surgery		●		Experimental	●					56%		●	●				●	
272	Pati	2014	ED	●			Qualitative					●	52%		●					●	
273	Pati	2015	ED			●	Observational					●	85%		●	●		●		●	●
274	Pati	2015	Medical		●		Observational					●	53%	●	●	●	●			●	
275	Pati	2016	ED	●	●		Qualitative			●			62%		●					●	
276	Pati	2016	Surgery		●		Experimental					●	56%			●			●		
277	Pati	2018	Medical		●		Observational					●	94%		●	●			●		
278	Patterson	2018	Medical	●			Qualitative				●		71%	●	●	●			●		●
279	Pearson	2018	Pediatric		●		Experimental					●	88%		●	●	●		●		
280	Perez-Urrestarazu	2017	General		●		Observational			●			94%	●		●	●		●	●	●
281	Persson	2015	Surgery	●			Qualitative				●		85%		●	●		●	●		
282	Persson Waye	2013	ICU		●		Experimental		●				90%	●					●		
283	Pettit	2014	ICU		●		Observational					●	84%		●				●		
284	Pickup et al.	2019	ED			●	Observational					●	62%		●				●		
285	Pineda	2012	PICU/NICU		●		Observational			●			63%		●				●		
286	Pink et al.	2020	General	●			Qualitative			●			62%	●	●	●	●	●		●	
287	Plough	2018	Maternal			●	Observational					●	54%	●	●	●		●		●	
288	Pouyesh	2018	Medical		●		Experimental			●			69%	●		●			●		
289	Price et al.	2018	Psychiatric	●			Qualitative					●	68%		●				●		
290	Pyrke	2017	Psychiatric		●		Observational			●			65%		●				●		
291	Quan	2012	Laboratory		●		Observational			●			59%	●		●			●	●	
292	Raanaas	2012	PICU/NICU		●		Observational				●		63%					●	●		
293	Raanaas	2015	Rehabilitation	●	●		Qualitative			●			67%		●	●		●	●		
294	Raiskila	2017	Pediatric		●		Observational				●		78%		●					●	
295	Rashid	2016	ICU		●		Observational	●					86%		●	●	●			●	
296	Rashid	2018	ICU		●		Observational			●			77%		●		●			●	
297	Real	2017	ED			●	Observational	●					67%		●					●	
298	Real	2018	Surgery			●	Observational				●		57%	●	●		●		●	●	
299	Real et al.	2019	General	●			Qualitative	●					57%		●	●	●			●	
300	Redstone	2011	Medical		●		Observational			●			65%			●			●		
301	Riet	2014	Pediatric	●			Qualitative			●			57%		●	●	●	●		●	
302	Robinson	2015	Palliative	●			Qualitative			●			62%	●	●	●	●		●		
303	Rodrigues et al.	2019	Pediatric	●			Qualitative		●				62%	●						●	
304	Rogers	2013	General	●			Qualitative					●	52%		●	●				●	
305	Rogers	2016	Psychiatric		●		Observational			●			70%	●	●	●	●		●	●	●

nr	Author	Year	Settings	Qualitative	Quantitative	Mixed methods	Research Design	Activity & behaviour	Clinical outcomes	Emotional wellbeing	PCC	Safe care	Quality	Ambient	Architectural	Interior	Social	Nature	Patients	Personell	Visitors
306	Rosbergen	2017	Medical	●			Qualitative			●			81%		●	●	●			●	
307	Rousek	2011	General		●		Experimental		●				50%	●	●	●					●
308	Sadatsafavi	2015	ED		●		Observational			●			55%	●	●	●		●		●	
309	Sadatsafavi	2015	General		●		Observational			●			60%	●					●		
310	Sadatsafavi	2015	ED		●		Observational			●			82%	●	●	●	●			●	
311	Sagah Zadeh et al.	2018	General			●	Observational					●	56%	●						●	
312	Sagha Zadeh	2018	General			●	Observational					●	58%	●	●	●	●	●	●		
313	Salgado	2013	ICU		●		Experimental					●	64%			●			●		
314	Sandal	2017	General			●	Experimental	●					93%	●	●	●	●	●	●	●	
315	Schreuder	2016	Medical		●		Observational			●			50%	●	●	●	●	●	●		
316	Scrivener et al.	2019	Rehabilitation		●		Observational	●					58%	●	●		●	●	●		
317	Shah	2011	Laboratory		●		Experimental	●					60%		●				●	●	
318	Shannon	2018	Rehabilitation		●		Observational	●					73%		●		●		●		
319	Shattell	2015	Psychiatric	●			Qualitative				●		62%		●				●	●	
320	Sheehan	2013	Psychiatric		●		Observational				●		54%		●			●		●	
321	Shepley	2016	Psychiatric	●			Qualitative				●		65%		●	●		●		●	
322	Shepley	2017	Psychiatric		●		Observational	●					53%	●	●	●	●	●		●	
323	Sherman-Bien	2011	Pediatric		●		Observational				●		75%	●	●	●		●	●		●
324	Shields et al.	2019	Psychiatric		●		Observational				●		64%			●			●		
325	Siddiqui	2015	Medical		●		Observational				●		82%	●	●	●			●		
326	Simon	2016	ED		●		Observational					●	67%		●				●		
327	Singh	2015	Medical		●		Observational					●	83%		●				●		
328	Sivak	2012	Psychiatric		●		Observational			●			52%	●		●			●		
329	Sjolander et al.	2019	General		●		Experimental		●				58%	●	●	●		●	●		
330	Skubik-Peplaski	2016	Rehabilitation			●	Experimental	●					53%	●	●	●	●			●	
331	Slatyer	2015	ED	●			Qualitative				●		71%	●	●	●	●			●	●
332	Smith	2016	Pediatric			●	Observational					●	69%		●	●	●			●	
333	Smith	2018	PICU/NICU		●		Observational					●	64%	●					●		
334	Sohrabi	2010	ED		●		Observational				●		57%		●		●			●	
335	Soremekun	2014	ED		●		Observational					●	100%		●				●		
336	Soubra et al.	2018	Pediatric		●		Observational				●		64%	●	●	●			●		
337	Southard	2012	Psychiatric		●		Observational			●			57%		●	●	●		●	●	
338	Steinke	2015	ED		●		Observational			●			74%	●	●	●	●			●	
339	Stevens	2010	PICU/NICU		●		Observational			●			65%	●	●	●	●	●		●	
340	Stevens	2011	Pediatric		●		Observational				●		59%	●	●	●	●	●			●
341	Stickland	2016	Pediatric	●			Qualitative			●			90%	●					●		●
342	Stiffler	2015	ED		●		Observational			●			70%		●				●		
343	Stiller	2017	ICU		●		Observational					●	57%	●	●	●				●	
344	Stremler	2011	Pediatric	●			Qualitative		●				86%	●	●	●	●		●		●
345	Sundberg	2017	ICU	●			Qualitative			●			76%	●	●	●	●	●		●	
346	Taghizadeh	2015	Maternal	●			Qualitative				●		52%			●			●		
347	Tandberg et al.	2019	PICU/NICU		●		Observational				●		62%	●	●	●	●		●		●
348	Tandberg et al.	2018	PICU/NICU		●		Observational	●					64%		●	●	●		●	●	●
349	Teltsch	2011	ICU		●		Observational					●	89%		●	●			●		
350	Thiel	2014	Pediatric		●		Observational				●		58%	●	●	●			●	●	
351	Thyssen	2014	Medical	●			Qualitative				●		71%		●	●			●		
352	Timmermann	2013	Medical	●			Qualitative			●			71%	●	●	●		●	●		
353	Timmermann	2015	Medical	●			Qualitative			●			76%	●	●	●	●	●	●		
354	Tinner	2018	Medical		●		Observational			●			60%	●	●	●	●	●	●	●	
355	Trevisani	2010	Medical		●		Observational			●			59%			●			●		
356	Trickey	2012	PICU/NICU		●		Observational		●				100%	●	●	●			●	●	
357	Trivedi et al.	2018	General		●		Observational				●		84%	●	●	●			●		
358	Trochelman	2012	ICU	●	●		Qualitative		●				71%	●	●	●	●	●	●		
359	Trudel et al.	2018	PICU/NICU	●			Qualitative					●	72%		●	●				●	
360	Trzpuc	2016	Psychiatric			●	Observational					●	70%	●	●	●	●	●	●	●	
361	Ullán	2012	Laboratory			●	Observational			●			87%	●	●	●	●		●	●	●
362	Ulrich	2018	Psychiatric		●		Observational	●					50%	●	●	●	●	●	●		
363	Ulrich et al.	2019	ICU		●		Observational		●				58%	●	●	●	●	●			●
364	Urbanoski	2013	Psychiatric		●		Observational				●		70%		●		●		●		
365	Vaisman	2018	Medical		●		Observational					●	89%		●				●		
366	van den Berg	2017	PICU/NICU		●		Observational				●		71%	●	●	●				●	

nr	Author	Year	Settings	Qualitative	Quantitative	Mixed methods	Research Design	Activity & behaviour	Clinical outcomes	Emotional wellbeing	PCC	Safe care	Quality	Ambient	Architectural	Interrior	Social	Nature	Patients	Personell	Visitors
367	van der Riet	2017	Pediatric	•			Qualitative			•			90%		•	•	•	•	•	•	
368	van der Riet et al.	2020	Pediatric	•			Qualitative				•		88%		•	•	•	•	•		
369	van der Schaaf	2013	Psychiatric		•		Observational					•	72%	•	•	•	•	•	•		
370	van Heuvelen	2019	PICU/NICU	•			Qualitative	•					68%	•	•	•	•			•	
371	Varughese	2012	Pediatric		•		Observational					•	76%		•	•			•		•
372	Venkatesh	2011	ED		•		Observational					•	73%		•					•	
373	Verceles	2013	ICU		•		Observational					•	68%	•	•				•		
374	Vesely	2017	Pediatric			•	Observational					•	56%	•	•	•	•		•	•	
375	Vokurka	2014	Medical		•		Observational					•	68%		•		•		•		
376	von Dessauer	2016	Pediatric		•		Experimental					•	89%			•			•		
377	Walsh	2010	ED		•		Observational				•		55%	•		•	•	•	•		
378	Wang	2013	ICU			•	Observational		•				63%	•	•	•				•	•
379	Wang	2017	Medical			•	Observational				•		53%	•	•	•	•	•	•		
380	Wang	2018	Medical			•	Observational		•				70%		•		•	•	•	•	
381	Wang et al.	2019	Surgery		•		Experimental		•				72%	•	•				•		
382	Warren	2013	Geriatric		•		Observational					•	84%			•			•		
383	Water	2017	Pediatric	•			Qualitative	•					61%	•	•	•		•	•		
384	Watkins	2011	Surgery		•		Observational				•		71%	•	•				•	•	
385	Watson	2014	PICU/NICU		•		Observational			•			69%	•	•		•			•	•
386	Watson	2015	Pediatric		•		Observational					•	68%	•						•	
387	Watson et al.	2019	General	•			Qualitative					•	72%	•	•	•			•		
388	Weldon	2015	Surgery			•	Observational				•		77%	•						•	
389	Wijk et al.	2019	Psychiatric		•		Observational				•		72%	•	•	•	•	•	•		
390	Williams	2011	ICU		•		Observational					•	66%			•			•		
391	Wingler	2015	General		•		Observational				•		68%	•	•	•				•	
392	Wingler & Keys	2019	General	•			Qualitative					•	72%	•	•	•				•	
393	Winner-Stoltz et al.	2018	PICU/NICU		•		Observational	•					78%	•	•	•	•			•	
394	Wright, Sarah	2019	General	•			Qualitative				•		62%	•	•	•	•				•
395	Wunsch	2011	ICU		•		Observational		•				72%	•	•				•		
396	Xidous et al.	2020	ED			•	Observational				•		58%	•	•	•	•		•		•
397	Xuan et al.	2019	General			•	Observational					•	62%		•		•			•	
398	Xuan et al.	2019	General			•	Observational	•					76%		•	•				•	
399	Ye et al.	2019	ED		•		Observational		•				58%	•					•		
400	Yildirim	2016	Surgery		•		Observational				•		57%	•	•	•	•		•		
401	Zaal	2013	ICU		•		Observational					•	75%	•	•				•		
402	Zadeh	2014	ED			•	Experimental					•	74%	•	•					•	
403	Zamani	2018	ED			•	Observational			•			50%	•	•	•	•			•	
404	Zborowsky	2010	Surgery			•	Observational	•					76%	•	•	•	•			•	
405	Zemni et al.	2018	General		•		Observational				•		69%	•	•				•		
406	Zhou	2016	General	•			Qualitative				•		71%		•	•			•	•	
407	Zisberg	2016	ED		•		Observational		•				83%		•	•			•		
